# Blocking the CTLA-4 and PD-1 pathways during pulmonary paracoccidioidomycosis improves immunity, reduces disease severity, and increases the survival of infected mice

**DOI:** 10.3389/fimmu.2024.1347318

**Published:** 2024-03-04

**Authors:** Nycolas Willian Preite, Bruno Montanari Borges, Valéria de Lima Kaminski, Marina Caçador Ayupe, Leonardo Mandu Gonçalves, Bianca Vieira dos Santos, Dennyson Leandro M. Fonseca, Igor Salerno Filgueiras, Caio Loureiro Salgado, Sandra Marcia Muxel, Otavio Cabral-Marques, Denise Morais da Fonseca, Flávio Vieira Loures, Vera Lúcia Garcia Calich

**Affiliations:** ^1^ Institute of Science and Technology, Federal University of São Paulo, São Paulo, Brazil; ^2^ Department of Immunology, Institute of Biomedical Sciences, University of São Paulo (USP), São Paulo, Brazil; ^3^ Institute of Mathematics and Statistics (IME), University of Sao Paulo (USP), Sao Paulo, Brazil; ^4^ Department of Medicine, Division of Molecular Medicine, University of São Paulo School of Medicine (USP), São Paulo, Brazil; ^5^ Department of Clinical and Toxicological Analyses, School of Pharmaceutical Sciences, University of São Paulo (USP), São Paulo, Brazil; ^6^ Network of Immunity in Infection, Malignancy, Autoimmunity (NIIMA), Universal Scientific Education and Research Network (USERN), São Paulo, Brazil

**Keywords:** pulmonary paracoccidioidomycosis, PD-1 and CTLA-4 blockade, protective effect, fungal clearance, reduced dissemination, improved immunity, increased survival, T cell responses

## Abstract

Immune checkpoint pathways, i.e., coinhibitory pathways expressed as feedback following immune activation, are crucial for controlling an excessive immune response. Cytotoxic T lymphocyte antigen-4 (CTLA-4) and programmed cell death protein-1 (PD-1) are the central classical checkpoint inhibitory (CPI) molecules used for the control of neoplasms and some infectious diseases, including some fungal infections. As the immunosuppression of severe paracoccidioidomycosis (PCM), a chronic granulomatous fungal disease, was shown to be associated with the expression of coinhibitory molecules, we hypothesized that the inhibition of CTLA-4 and PD-1 could have a beneficial effect on pulmonary PCM. To this end, C57BL/6 mice were infected with *Paracoccidioides brasiliensis* yeasts and treated with monoclonal antibodies (mAbs) α-CTLA-4, α-PD-1, control IgG, or PBS. We verified that blockade of CTLA-4 and PD-1 reduced the fungal load in the lungs and fungal dissemination to the liver and spleen and decreased the size of pulmonary lesions, resulting in increased survival of mice. Compared with PBS-treated infected mice, significantly increased levels of many pro- and anti-inflammatory cytokines were observed in the lungs of α-CTLA-4-treated mice, but a drastic reduction in the liver was observed following PD-1 blockade. In the lungs of α-CPI and IgG-treated mice, there were no changes in the frequency of inflammatory leukocytes, but a significant reduction in the total number of these cells was observed. Compared with PBS-treated controls, α-CPI- and IgG-treated mice exhibited reduced pulmonary infiltration of several myeloid cell subpopulations and decreased expression of costimulatory molecules. In addition, a decreased number of CD4+ and CD8+ T cells but sustained numbers of Th1, Th2, and Th17 T cells were detected. An expressive reduction in several Treg subpopulations and their maturation and suppressive molecules, in addition to reduced numbers of Treg, TCD4+, and TCD8+ cells expressing costimulatory and coinhibitory molecules of immunity, were also detected. The novel cellular and humoral profiles established in the lungs of α-CTLA-4 and α-PD-1-treated mice but not in control IgG-treated mice were more efficient at controlling fungal growth and dissemination without causing increased tissue pathology due to excessive inflammation. This is the first study demonstrating the efficacy of CPI blockade in the treatment of pulmonary PCM, and further studies combining the use of immunotherapy with antifungal drugs are encouraged.

## 1 Introduction

A balance of costimulatory and coinhibitory signals tightly controls T-cell activation by antigen-presenting cells (APCs). Immune checkpoint pathways, i.e., coinhibitory pathways expressed as feedback following immune activation or in response to chronic antigen exposure and inflammation, are crucial for inducing tolerance to self-antigens, limiting autoimmunity, and controlling an excessive immune response (1).. However, checkpoint pathways also promote immune exhaustion in scenarios of excessive or chronic antigen exposure due to inflammation, infection, or cancer (1–3). Although the exact definition of immune exhaustion is debated, the term generally refers to a hypofunctional state of immune cells, especially T cells, characterized by reduced cytokine production, limited proliferation, epigenetic and metabolic changes, and upregulation of inhibitory receptors (4).

Cytotoxic T lymphocyte antigen-4 (CTLA-4) and programmed cell death protein-1 (PD-1) are the main classical checkpoint pathways associated with immune exhaustion. The CTLA-4 pathway predominantly activates naïve T cells at the priming stage. The most potent costimulatory signal for naïve T-cell activation is mediated by the interaction of CD28 molecules on the T-cell membrane with CD80 and CD86 on antigen-presenting cells (APCs). To prevent excessive activation and self-reactivity, the CD28 pathway is downregulated through the expression of CTLA-4, which then overrides the interaction of CD28 with CD80 and CD86 due to its increased affinity, resulting in inhibitory signals (1–3, 5). Couples of the molecules CD154-CD40, OX40-OX40L, and 4-1BB-4-1BBL also cooperate in costimulatory processes (6, 7).

PD-1 is most widely expressed in T cells, B cells, natural killer cells (NKs), and mononuclear phagocytes and is rapidly activated upon T-cell receptor (TCR) stimulation. PD-1 signaling inhibits T-cell survival and proliferation, suppresses T-cell effector cytokine release, and interferes with TCR signaling. PD-1 interacts with two major ligands, PD-L1 and PD-L2, which are widely expressed by professional APCs as well as nonlymphoid cells and tissues (2, 3, 8, 9). IFN-γ, which is secreted by activated T cells, is considered the most potent stimulus for PD-L1 upregulation (9). In addition to these classic immunological checkpoints, other molecules with coinhibitory functions are being increasingly studied (10).

Several reports have documented the activation of inhibitory checkpoint pathways in fungal infections (6, 7, 11). There is good documentation of immune system exhaustion and immune checkpoint pathway induction, mainly during opportunistic fungal infections but also for other endemic dimorphic fungi. In addition, some preclinical studies suggest the protective therapeutic effect of CPI blockade in murine models of fungal infections (7, 12). However, there are still significant knowledge gaps in this area, indicating the expansion of experimental and preclinical studies in fungal infections (7).

The immune response against PCM, a primary systemic mycosis caused by the dimorphic fungus *Paracoccidioides brasiliensis*, which is endemic in Latin America, is strongly influenced by disease manifestations. The acute form of the disease is characterized by the predominant activation of Th2/Th9 cells, and patients with the chronic form develop a mixed immune response with predominant differentiation of Th1/Th17/Th22 cells and high production of IL-17 and IL-22. Alternatively, individuals with asymptomatic infections without disease develop more polarized Th1 immunity (13). The disease can be characterized by a chronic course and the involvement of multiple organs; pulmonary failure; and Addison’s syndrome, which affects the adrenal glands and is the main sequelae. The treatment of mild forms is usually performed with fast-acting azoles and sulfas. Severe forms, whether acute or chronic, involve complex and long-term treatment; an initial approach involving amphotericin B is often used. Therefore, itraconazole is the first choice for maintenance treatment. However, in patients involving the central nervous or digestive systems or who use proton pump inhibitors, itraconazole is not recommended due to limited blood-brain barrier penetration and reduced absorption, respectively. Consequently, itraconazole may not always be employed in more serious cases, and additional extensive studies and new therapeutic approaches are needed (14).

Since the 1970s, the severity of PCM has been associated with suppressed T-cell responses (15, 16). The mechanisms involved in this immunosuppression are still not fully understood but seem to be related to the unbalanced production of suppressor cytokines (IL-10 and TGF-β) and membrane-bound inhibitory mediators (CTLA-4 and PD-1), suggesting an essential role for regulatory T cells (Tregs) in the development and control of PCM (17–20).

Treg cells expressing the Foxp3 transcription factor (TregFoxp3+) are regularly detected in inflammatory lesions and in the peripheral blood of patients with PCM. The number of these cells correlates with the density of *P. brasiliensis* cells in the lesions and with the severity of the disease (19–21). Human Treg cells isolated from PCM patients have been shown to depend on CTLA-4-mediated cell-cell contact and the production of anti-inflammatory cytokines (IL-10 and TGF-β) (20, 21).

Experimental models have expanded the knowledge of immunoregulation in PCM and provided support for human studies. Indeed, our findings on the role of Treg cells in pulmonary PCM demonstrated the dual role of this T-cell subpopulation (22). Although Treg cell depletion increases host resistance mediated by Th1 and Th17 cells, the total absence of these cells leads to excessive immunity and increased tissue pathology. In the course of lung disease, infected mice develop increasing numbers of Treg cells that express LAP (membrane TGF-β), the inhibitory molecules CTLA-4 and PD-1, in addition to intracellular TGF-β and IL-10 (23, 24). These immune inhibitory molecules are expressed by both conventional T lymphocytes (CD4+ Foxp3-) and Treg cells (CD4+ CD25+ Foxp3+) but are expressed at higher levels by Treg cells (22, 24, 25). Notably, the severity of murine PCM is also affected by the presence of myeloid-derived suppressor cells (MDSCs), which contribute to the suppressive microenvironment in the lung by producing immunosuppressive molecules that disrupt the activation of T lymphocytes (26, 27).

Our studies also showed the importance of the enzyme indoleamine 2,3-dioxygenase-1 (IDO-1) and the transcription factor aryl hydrocarbon receptor (AhR) in the immunoregulation of PCM. Using several experimental approaches, we demonstrated that IDO expands Treg cells (28–31) and that AhR plays an essential role in the development of Th17, Tr1, and Treg cell subpopulations (32, 33).

In summary, experimental and clinical data indicate that checkpoint molecules play essential roles in the immunosuppressive mechanisms of PCM. In addition to the consistent demonstration of CTLA-4 and PD-1 participation in the immunosuppression associated with the severe forms of human and experimental PCM (17, 18, 20–24), the successful use of checkpoint inhibitors for the control of neoplasms (34) and some fungal infections (6, 7) led us to postulate that the inhibition of these molecules could have a beneficial effect on PCM. Most importantly, PCM treatment is difficult and prolonged, and there are few available antifungals that cause serious side effects and prevent patient adherence to treatment. Therefore, in this work, we analyzed the effects of CTLA-4 and PD-1 blockade on the fate of the disease and the immune response in a murine model of pulmonary PCM. We verified that inhibiting these molecules reduces the fungal load, tissue pathology, and mortality in mice. The blockade of CPI molecules rescues efficient protective immunity that controls fungal growth and dissemination without exacerbating deleterious inflammatory processes. This is the first study showing the beneficial effect of CPI blockade for pulmonary PCM, indicating the possibility of using anti-CPI treatment in combination with antifungal drugs as an adjunctive therapy.

## 2 Materials and methods

### 2.1 Ethics statement

The experiments were performed in strict accordance with the Brazilian Federal Law 11,794, establishing procedures for the scientific use of animals, and the State Law establishing the Animal Protection Code of the State of São Paulo. All efforts were made to minimize animal suffering. The procedures were approved by the Ethics Committee on Animal Experiments of the Institute of Biomedical Sciences of the University of São Paulo (Proc.180/11/CEEA).

### 2.2 Mice

C57B/6 SPF male mice, bred at the Isogenic Breeding Unit of the Department of Immunology, Institute of Biomedical Sciences, were used at the age of 8-12 weeks.

### 2.3 Fungus and intratracheal infection

The virulent Pb18 isolate from *P. brasiliensis* was maintained in the yeast form by weekly cultivation in Fava Netto’s semi-solid medium at 36°C and used on days 6–8 of culture. The fungus was collected and washed with phosphate-buffered saline (PBS, pH 7.2). The fungal viability was determined by the Janus Green B vital dye. All experimental procedures were carried out with fungal suspensions presenting viability between 90 and 95%. For it. infection, mice were anesthetized with ketamine (90 mg/kg) and xylazine (10 mg/kg) and submitted to it. infection with 1x10^6^ yeast cells in 50 μL of PBS as previously described (35). Mice were euthanized under anesthesia by ip. injection of xylazine (30 mg/kg) and ketamine (180 mg/kg) at week 8 post-infection, or throughout the course of infection if they presented clinical signs of pain/stress, such as loss of appetite and heaviness, arching of the spine, low activity or bristly hair.

### 2.4 Treatments with α-CTLA-4, α-PD-1, control IgG mAbs or PBS

Three weeks after *P. brasiliensis* infection, groups of mice received three times a week intraperitoneal (ip.) injections of 200 μg contained in 100 μl of mAb α-CTLA-4 (BioXCell Cat # BE0032 clone UC10F10-11, hamster IgG)), α-PD-1 (CD279, BioXCell, Cat # BE0146, clone RMP1-14, rat IgG2a), control monoclonal IgG ((BioXCell, Cat # BE0089, clone 2A3, rat IgG2a), or 100 μl of PBS (Phosphate Buffered Saline). All mAbs contained <2EU/mg of endotoxin (<0.002EU/μg) as determined by LAL gel clotting assay. The dose of α-CTLA-4 and α-PD-1 mAbs corresponds to approximately 10 μg/kg of body weight. Treatment was started in the third week post-infection because in this period the adaptive immunity and the expansion of its regulatory mechanisms, such as the expression of CTLA-4 and PD-1, are already established in infected C57BL/6 mice (23–26). The treatment was maintained for five weeks. Animals were sacrificed eight weeks after infection, and the immune response and disease severity were analyzed by various methods. The experiments were repeated twice.

### 2.5 CFU assays, mortality rates, and histological analysis

The number of viable yeasts in the infected organs (lung, liver, and spleen) was determined by counting the number of colony-forming units (CFU), as previously described (36). Survival studies were carried out with groups of 10 to 12 mice. Deaths were recorded daily. For histological examinations, the left lung of infected mice was removed and fixed in 10% formalin. Five-micrometer sections were stained with HE for lesion analysis and silver (Grocott) for fungal evaluation. Morphometric analysis was performed using a Nikon DXM 1200c camera and Nikon NIS AR 2.30 software.

### 2.6 Detection of cytokines in lung and liver cell supernatants

The lungs and liver of treated and control mice were collected eight weeks after *P. brasiliensis* infection. Organs were aseptically removed and individually dissociated in 5 mL of PBS. The supernatants were separated and stored at -80° C. Levels of IL-23, IL-6, IL-17, IL-22, IL-12, IFN-γ, IL-2, GM-CSF, IL-5, IL-4, IL-10, and TGF-β were measured by enzyme-linked immunosorbent assay (ELISA) capture with antibody pairs purchased from eBioscience. Plates were read using a spectrophotometric plate reader (VersaMax, Molecular Devices).

The heatmap of cytokines was generated using the ComplexHeatmap (37) and Circlize (38) libraries using the R programming language (The R Project for Statistical Computing https://www.r-project.org/) with the assistance of the RStudio (R-Studio https://www.rstudio.com/) interface. For this purpose, the individual values of 12 markers (cytokines) were transformed into z-scores based on the mean for each marker in two tissues (Liver and Lung). The dendrograms were constructed based on the Euclidean distance (37, 39) clustering methodology. In addition, for better visualization, the clustering was performed only within each group for each tissue.

### 2.7 Assessment of pulmonary leukocyte subpopulations by flow cytometry

Lungs were removed shortly after euthanasia and processed in digestion buffer (RPMI medium (Sigma, St. Louis, MO, USA) containing 0.5 mg/mL DNAse I (Sigma-Aldrich, USA) and 1 mg/mL of collagenase IV (Sigma Aldrich, USA). Digestion was performed at 37°C for 30-40 min. Once homogenized, the digested samples were passed through 70 µm cell strainers and transferred to conical centrifuge tubes containing 10 mL of complete RPMI (3% FBS, Sigma-Aldrich, St. Louis, MO, USA, 10 mg/mL penicillin + 10,000 U/mL streptomycin (Hyclone, Logan, UT, USA), 0.3 g/mL L - glutamine (Sigma-Aldrich, USA), 0.0040 g/mL beta-mercaptoethanol (Sigma-Aldrich, USA), 0.0089 g/mL non-essential amino acids (Sigma-Aldrich, USA), 0.0089 g/mL sodium pyruvate (Sigma -Aldrich, USA), and then centrifuged at 4°C for 8 min at 1600 rpm. Supernatants were discarded, and cells were resuspended in 500 μL of RBC lysis buffer and incubated on ice. After 3 min incubation, cells were washed and resuspended in complete RPMI, counted, and prepared for cytometric analysis. Two million lung cells were stained for surface markers or transcription factors (**Supplementary Figure 1**). Intranuclear staining for transcription factors was performed according to the manufacturer’s instructions using an eBioscience Transcription Factor Buffer Set. In parallel, 2 million cells were used to detect intracellular cytokines. Cells were incubated for 6 h with 100 μL of complete RPMI containing 50 ng/mL phorbol myristate acetate (PMA) (Sigma Aldrich, USA), 500 ng/mL ionomycin (Sigma-Aldrich, USA), and 1 μL/mL from Golgi Plug (BD Biosciences, San Jose, CA, USA). In all different panels (**Supplementary Table 1**) employed, before adding the antibody mixture, all samples were incubated for 20 min at 4°C with 30 μL of Live/Dead, 1:1000 (LD, Thermo Fisher Scientific, USA), followed by surface staining and intracellular/intranuclear staining. Data were acquired on the BD LSRFortessa X-20 flow cytometer (BD Biosciences, USA), and data compensation and analysis were performed with FlowJo software. The gate strategies are described in **Supplementary Figures 1, 3**–**5**.

The following leukocytes populations were characterized: ILC1 (CD45+Live/Dead-CD90.2+Lin-NK1.1+or-NKp46+or-Tbet+), ILC2 (CD45+Live/Dead-CD90.2+Lin-GATA-3+ and IL-5+IL-13+), ILC3 (CD45+Live/Dead-CD90.2+Lin-NK1.1−NKp46−CD127+Tbet+/-RORγτ+ and IL-17+IL-22+), lymphocytes Tγδ (CD45+Live/Dead-CD90.2+CD3+TCRγδ+), Neutrophils (CD45+Live/Dead-CD11b+Ly6G+MHCII-), Inflammatory Monocytes (CD45+Live/Dead-CD11b+Ly6ChighLy6G-MHCII+), macrophages (CD45+Live/Dead-CD11b+CD64+F480+) and dendritic cells (CD45+Live/Dead-Lin-CD11c+MHCII+CD11b+/- and CD103+/-). The lymphocyte subpopulations were also characterized (CD45+Live/Dead-, CD90.2+CD3+CD4+, being TCRβ+ or TCRαβ+) Th1 (Tbet+), Th2 (GATA3+), Th17 (RORγτ+) and Treg (Foxp3+) and, finally, the CD8+ T lymphocytes (CD45+Live/Dead-CD90.2+TCRβ+CD8+).

### 2.8 Dimensionality reduction of flow cytometric data using the t-distributed stochastic neighbor embedding algorithm

In defined experiments, tissue-infiltrating leukocytes were characterized based on the expression of surface molecules using flow cytometry and the t-distributed Stochastic Neighborhood Embedding (t-SNE) algorithm for dimensionality reduction. To differentiate infiltrating parenchymal leukocytes from the associated vascular fraction, mice were injected intravenously with 3 µg of FITC-labeled anti-CD45 antibody (Biolegend, San Diego, CA, USA) in 200 µL of sterile saline. After 3 min, the mice were sacrificed, and the lungs were perfused and collected for tissue processing as described above. The vascular fraction of leukocytes was identified based on anti-CD45 FITC staining. Then algorithm analysis (t-SNE) was applied: 84,000 events per group were reduced from the total number of live and concatenated Thy1.2+ lymphoid cells. The t-SNE algorithm was applied to the concatenated samples using 2000 interactions and 80 perplexities. Then, the cell clusters were separated into vascular and stromal fractions (based on CD45-FITC staining), and the subsets of cells in each cluster were identified based on the gating strategy described in **Supplementary Figure 1**. The percentage of each subset of cells was calculated after segregating the groups based on the sample IDs and described in the table below the t-SNE representations.

### 2.9 Statistical analysis

Data are presented as the mean ± standard error of the mean (M ± SEM). The normality of the distribution was evaluated using the Kolmogorov-Smirnov test. Differences between groups were analyzed by non-paired Student’s t-test or analysis of variance (ANOVA) followed by the Tukey test. Differences between survival times were determined with the log-rank test. Data were analyzed using GraphPad Prism 9 software (GraphPad Prism Software, Inc., USA). *p* values ≤ 0.05 were considered significant.

## 3 Results

### 3.1 Treatment with α-CTLA-4 and α-PD-1 mAbs during the course of pulmonary PCM reduces the fungal burden on organs and the severity of pulmonary lesions and increases the survival of mice

**Figure 1:**
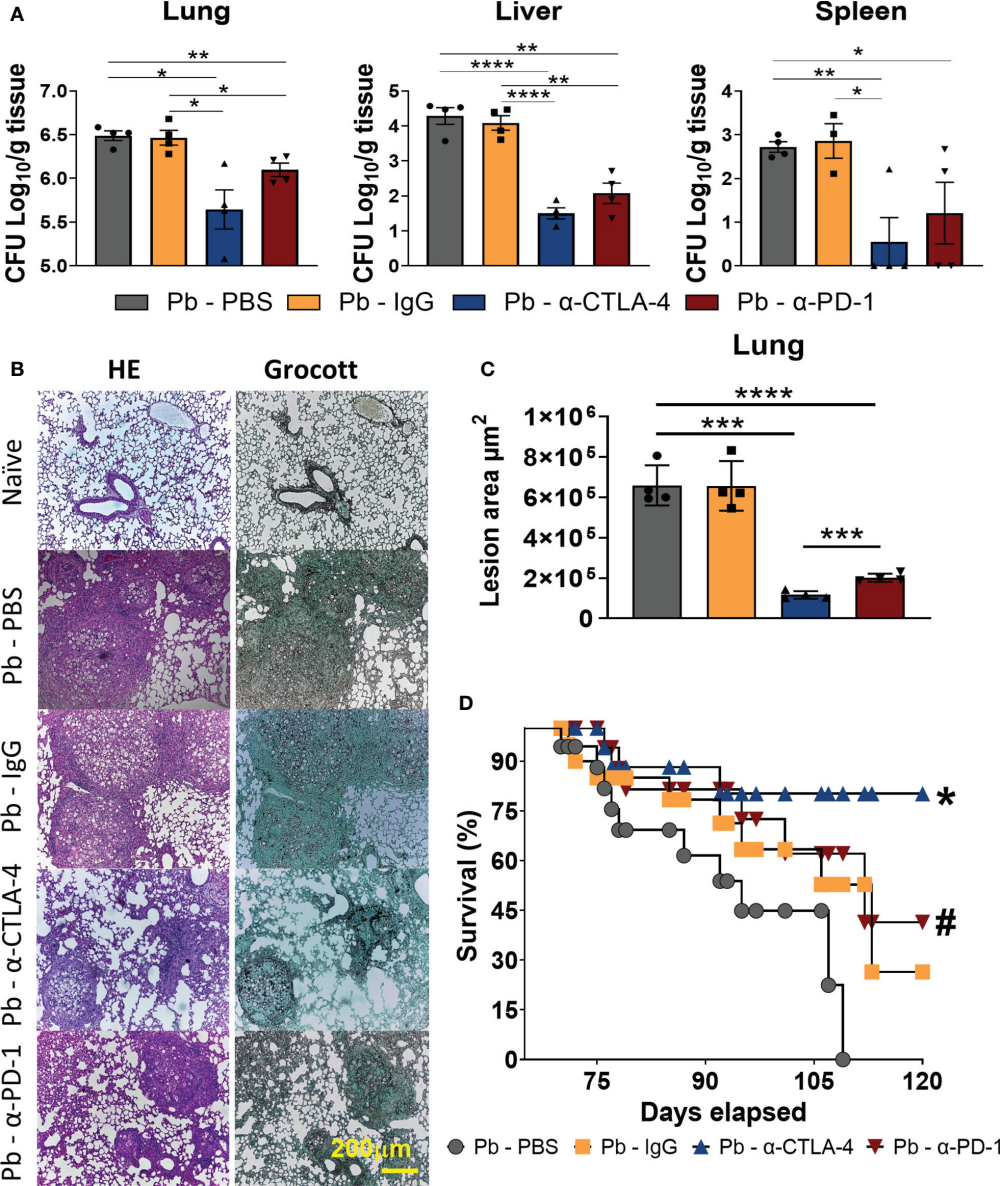
Treatment with α-CTLA-4 and α -PD-1 mAbs reduces fungal loads, tissue pathology, and increases survival of *P. brasiliensis* infected mice. C57BL/6 mice were infected with 1x10^6^ *P. brasiliensis* yeasts and 3 weeks later treated three times a week (for five weeks) with 200 μg of α-CTLA-4, α -PD-1, control IgG mAbs or PBS. The organs were obtained at the 8^th^ week post-infection (n: 4-7) and the number of viable fungi (CFU) present in the lungs, liver, and spleen measured **(A)**. At the 8^th^ p.i. week, the organs were removed, fixed, and histologically analyzed after tissue staining by Haematoxilin-Eosin (HE) or Grocott (Gro) and representative images are shown (yellow scale bar represents 200 µm) **(B)**. Tissue pathology was evaluated considering size, composition, and presence of fungi in inflammatory infiltrates. Lesion areas in the lungs were measured by morphometric analysis performed using a Nikon DXM 1200c camera and Nikon NIS AR 2.30 software **(C)**. Experiments were repeated twice using 4-5 mice per group, and results presented as mean +/- SEM. Differences were considered significant when: *p<0.05; **p<0.01; ***p<0.001 and ****p<0.0001. Animal mortality (n=12) was monitored daily **(D)** and survival time differences characterized using a LogRank test. *P* values < 0.05 were considered significant, * *p* < 0.006, and # *p* < 0.03 **(D)**.

C57BL/6 mice (n=4-7) were infected with 1x10^6^ *P. brasiliensis* yeast and, after 3 weeks, treated 3 times a week for 5 weeks with 200 µg of α-CTLA-4, α-PD-1, control IgG mAbs or PBS. Organs were obtained at week 8 post infection (p.i.), and the number of viable fungi (CFU) in the lungs, liver, and spleen was analyzed (**Figure 1A**). A significant decrease in viable fungal cells was observed in the lungs, liver, and spleen of α-CTLA-4- and α-PD-1-treated mice. Lungs obtained at week 8 p.i. were fixed, laminated, and stained with HE and Grocott. Both α-PD-1 and α-CTLA-4 reduced the severity of lung lesions that also contained fewer fungi (**Figure 1B**). Morphometric analysis also demonstrated that anti-CPI treatments reduced the area of lung lesions (**Figure 1C**). Survival was also analyzed in another group of mice (n=12), and both α-CTLA-4 and α-PD-1 treatments significantly reduced the mortality of infected animals (**Figure 1D**).

### 3.2 Treatment with α-CPIs increases the levels of pro- and anti-inflammatory cytokines in the lungs but drastically reduces liver cytokine levels

**Figure 2:**
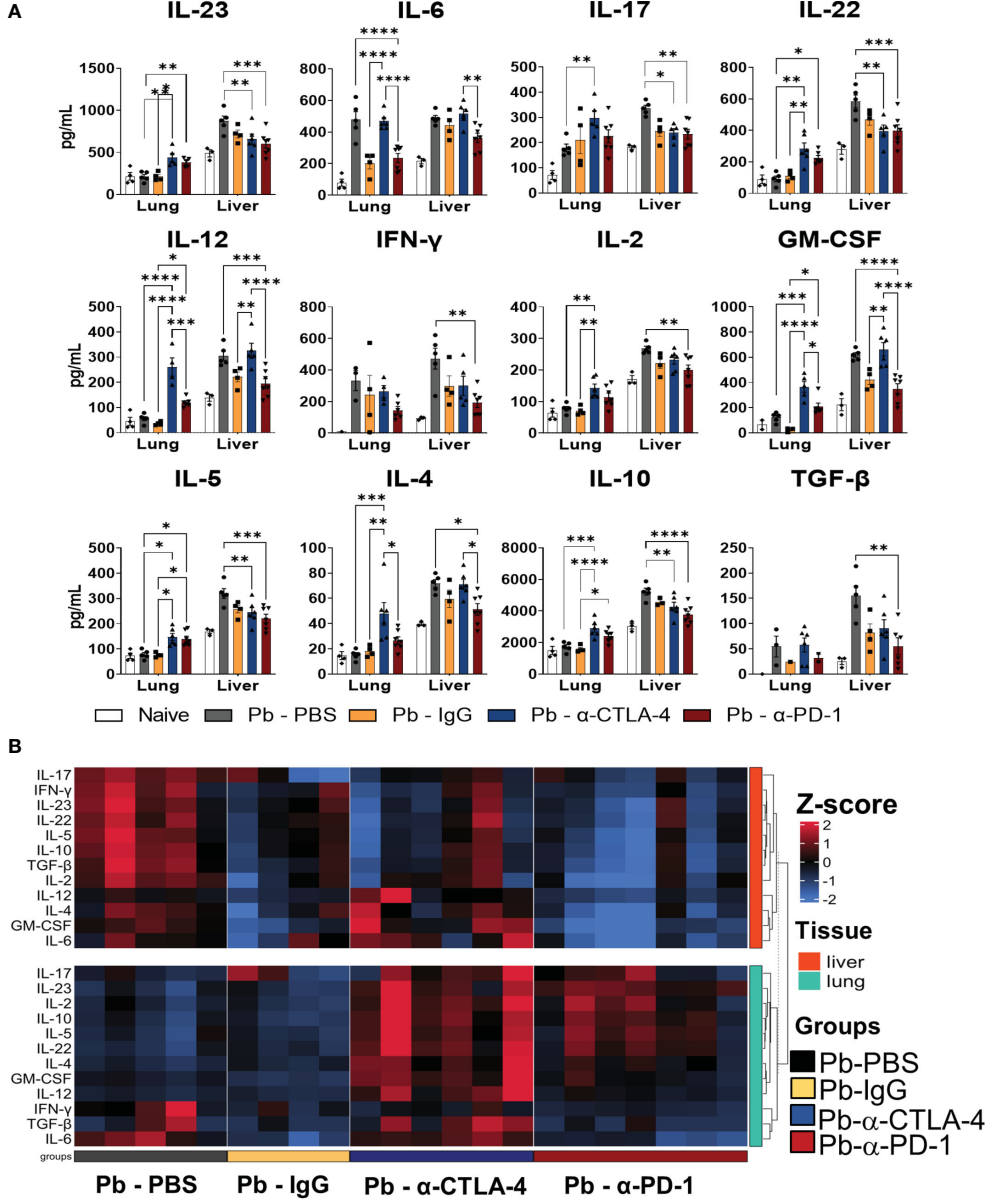
Treatment with checkpoint inhibitors increases the levels of pro- and anti-inflammatory cytokines in the lungs, but drastically reduces liver cytokines. Cytokines (IL-23, IL-6, IL-17, IL-22, IL-12, IL-2, IFN-γ, GM-CSF, IL-4, IL -5, IL-10 and TGF-β) in the lungs and liver supernatants of *P.brasiliensis* infected mice treated with a-CTLA-4, a-PD-1, control IgG mAbs or PBS were analyzed by ELISA. Experiments were repeated twice using 4-7 mice per group, and results presented as mean +/- SEM. Differences were considered significant when: *p<0.05; **p<0.01; ***p<0.001 and ****p<0.0001 **(A)**. The heatmap displays Z-score values ranging from -2 to 2, as indicated by the color scale bar on the side of the graph. The data was clustered based on Euclidean distance for each tissue, with rows corresponding to tissue types. The columns represent individual samples within their respective groups, and the rows represent twelve distinct cytokines. Data for liver cytokines are in the upper part (red), while lung cytokines in the lower part (green) of the heatmap **(B)**.

The lungs and livers obtained at the 8^th^ week p.i. were macerated, the supernatants were collected, and the cytokine levels were measured using capture ELISA. **Figure 2A** shows the levels of pro- and anti-inflammatory cytokines (IL-23, IL-6, IL-17, IL-22, IL-12, IFN-γ, IL-2, GM-CSF, IL-5, L-4, IL-10, and TGF-β) present in the lungs and liver of the different groups of mice studied. **Supplementary Table 2** summarizes the significant differences induced by both treatments.

Compared with Pb-PBS, α-CTLA4 treatment increased the levels of many pulmonary cytokines (IL-23, IL-17, IL-22, IL-12, IL-2, GM-CSF, IL-10, IL-4, and IL-5). Treatment with α-PD-1 increased IL-23, IL-22 and IL-5 levels but reduced IL-6 levels. In the liver, α-PD-1 therapy led to a drastic reduction in cytokines (IL-23, IL-17, IL-22, IL-12, IL-2, IFN−γ, GM-CSF, IL-10, TGF-β, IL-4, and IL-5), accompanied by a significant reduction in fungal loads. Treatment with α-CTLA-4 also reduced the presence of several hepatic cytokines (IL-23, IL-17, IL-22, IL-10, and IL-5) (**Figure 2A**; **Supplementary Table 2**).

Using Pb-IgG as a control, CTLA-4 blockade increased the levels of almost all pulmonary cytokines studied except for IL-17, IFN-γ, and TGF-β, whereas a negligible effect was observed in the liver (increased IL-12 and GM-CSF). Increased levels of IL-12, GM-CSF, IL-10, and IL-5 were detected in the lungs, but no differences were found in the livers of α-PD-1-treated mice (**Figure 2A**; **Supplementary Table 2**).

A heatmap of cytokines present in the lungs and livers of individual mice in each studied group is shown in **Figure 2B**. As such, unexpectedly, the treatment with control IgG caused a reduced presence of cytokines in the lungs and livers. It also exhibits the significant increase of pulmonary cytokines induced by α-CTLA-4 and the significative reduction in the livers caused by α-PD-1 treatment.

### 3.3 Compared with PBS-treated controls, α-CPI treatments significantly decreased the numbers of TCD4+, TDC8+ and Treg cells without reducing the Th1, Th2, or Th17 cell subsets in the lungs of infected mice

**Figure 3:**
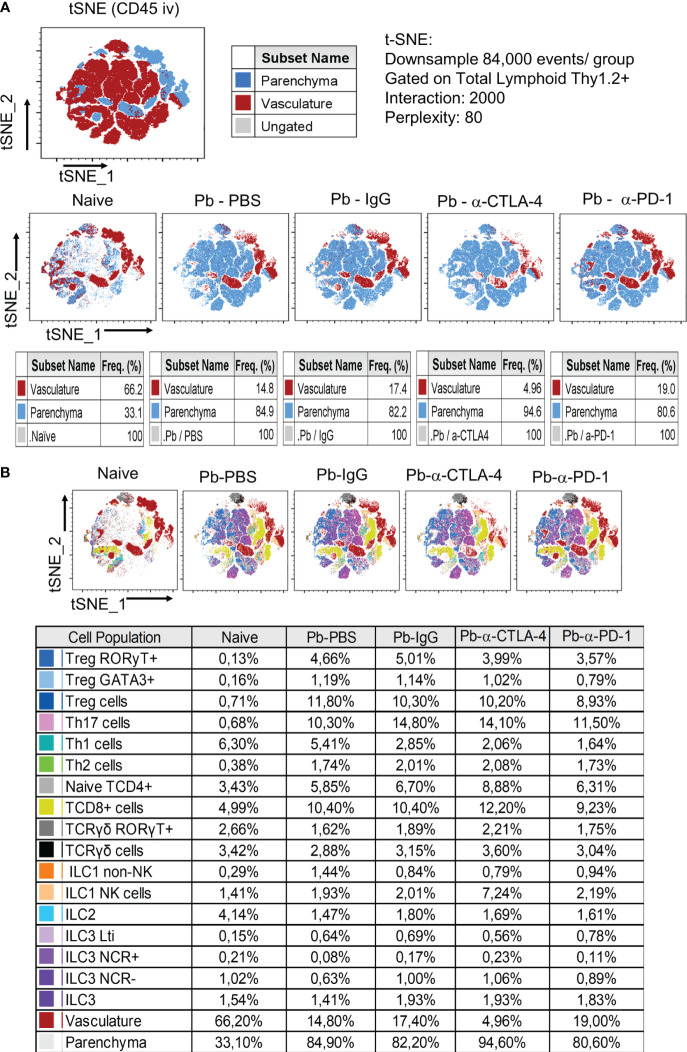
Analysis by tSNE (t-distributed Stochastic Neighbor Embedding) of CD45+ Lung Leukocytes. Pulmonary leukocytes were obtained at the 8^th^ week p.i. of mice with *P. brasiliensis* and treated during the course of the disease (3rd week p.i. on) with α-CTLA-4, α-PD1and control IgG mAbs or PBS for five weeks. Before sacrifice, the animals were inoculated ev. with fluorescein labeled α-CD45 antibodies. Total lung leukocytes were gated as Thy1.2+ and the frequency of vasculature and parenchymal cells characterized **(A)**. T and Innate Lymphoid Cells subpopulations present in the lungs of mice at the 8^th^ week p.i. were also characterized by tSNE analysis **(B)**. Experiments were repeated twice using 4-7 mice per group, and results presented as frequency of CD45+ lung cells.

**Supplementary Figure 1** shows the gating strategy used to analyze lung CD45+ cells by flow cytometry, and **Figure 3** shows the total numbers of several analyzed T lymphocyte subsets. Importantly, for greater clarity of the flow cytometry graphs, significant differences between the group of naïve mice and all groups of infected animals were not shown.

Like for cytokine levels, control IgG caused an essential difference in the migration of lung leukocytes. Compared with those of the Pb-PBS control, all the mAb treatments reduced the number of CD45+ leukocytes that migrated to the lungs. In contrast, minor differences in the frequencies of these cells were detected, although the Pb-α-CTLA-4 group exhibited an increased frequency of CD45+ cells compared with the Pb-IgG and Pb-α-PD-1 groups (**Figure 3**).

**Figure 4:**
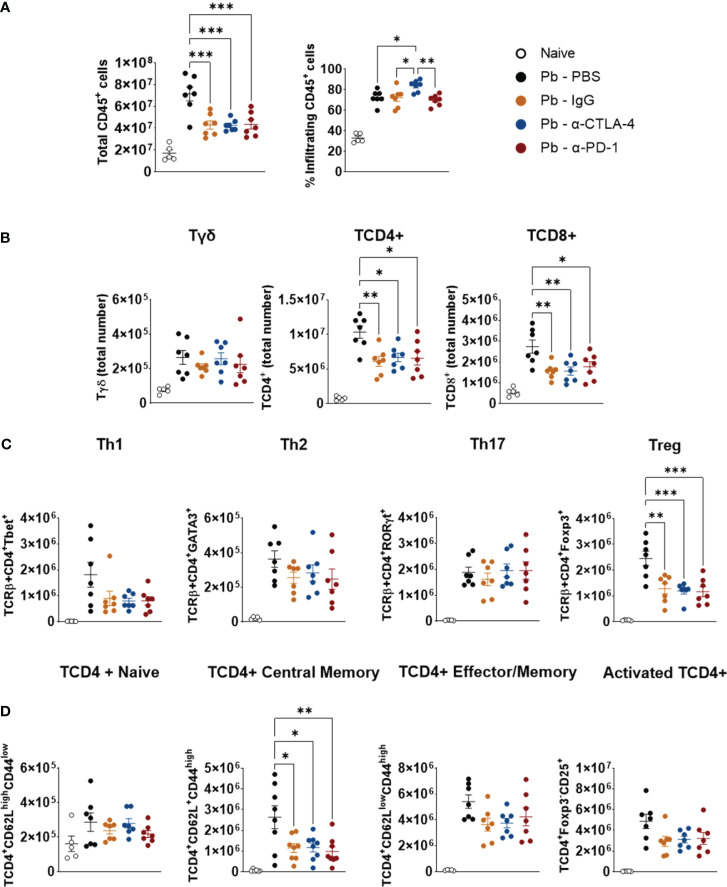
Compared with control Pb-PBS, checkpoint blockade significantly reduces total cell counts, TCD4+, TDC8+, Treg, and TCD4+ Central Memory subpopulations, without, however, reducing Th1, Th2, and Th17 subpopulations in the lungs of infected mice. Total lung cells, frequency and number of lungs infiltrating CD45+ leukocyte subsets were analyzed by flow cytometry **(A)**. Number of Tγδ, TCD4+, TCD8+ **(B)**, Th1, Th2, Th17, Treg **(C)**, and Naïve, Central Memory, Effector/Memory and Activated TCD4+ lymphocytes **(D)** present in the lungs of mice at the 8^th^ week post-infection with 1 x 10^6^ P*. brasiliensis* yeasts and treated with α -CTLA-4, α -PD-1, control IgG mAbs or PBS for 5 weeks. Experiments were repeated twice using 4-7 mice per group. Differences were considered significant when: *p<0.05; **p<0.01; and, ***p<0.001.

All the mAb treatments reduced the numbers of TCD4+, TCD8+, and Treg (CD4+Foxp3+) cells; however, no differences in the total numbers of Th1, Th2, or Th17 cells were detected, suggesting that the reduction in CD4+ cells occurred mainly at the expense of the decrease in Treg cells (**Figures 4A–C**). In addition, no differences were detected in the number of naïve, effector/memory, or activated CD4+ lymphocytes. However, diminished lung infiltration of CD4+ central memory cells (**Figure 4D**) was observed in the lungs of the mAb-treated mice. We also assessed the presence of innate lymphoid cells in the lungs at the 8^th^ p.i. week. Almost no differences were detected among the groups, as shown in **Supplementary Figure 2**.

### 3.4 Compared with PBS-treated controls, blockade of CPI molecules reduces the number of Treg cells expressing Tbet, GATA3, RORγτ, Neuropilin, Helios, and the suppressive ectoenzymes CD39 and CD73

**Figure 5:**
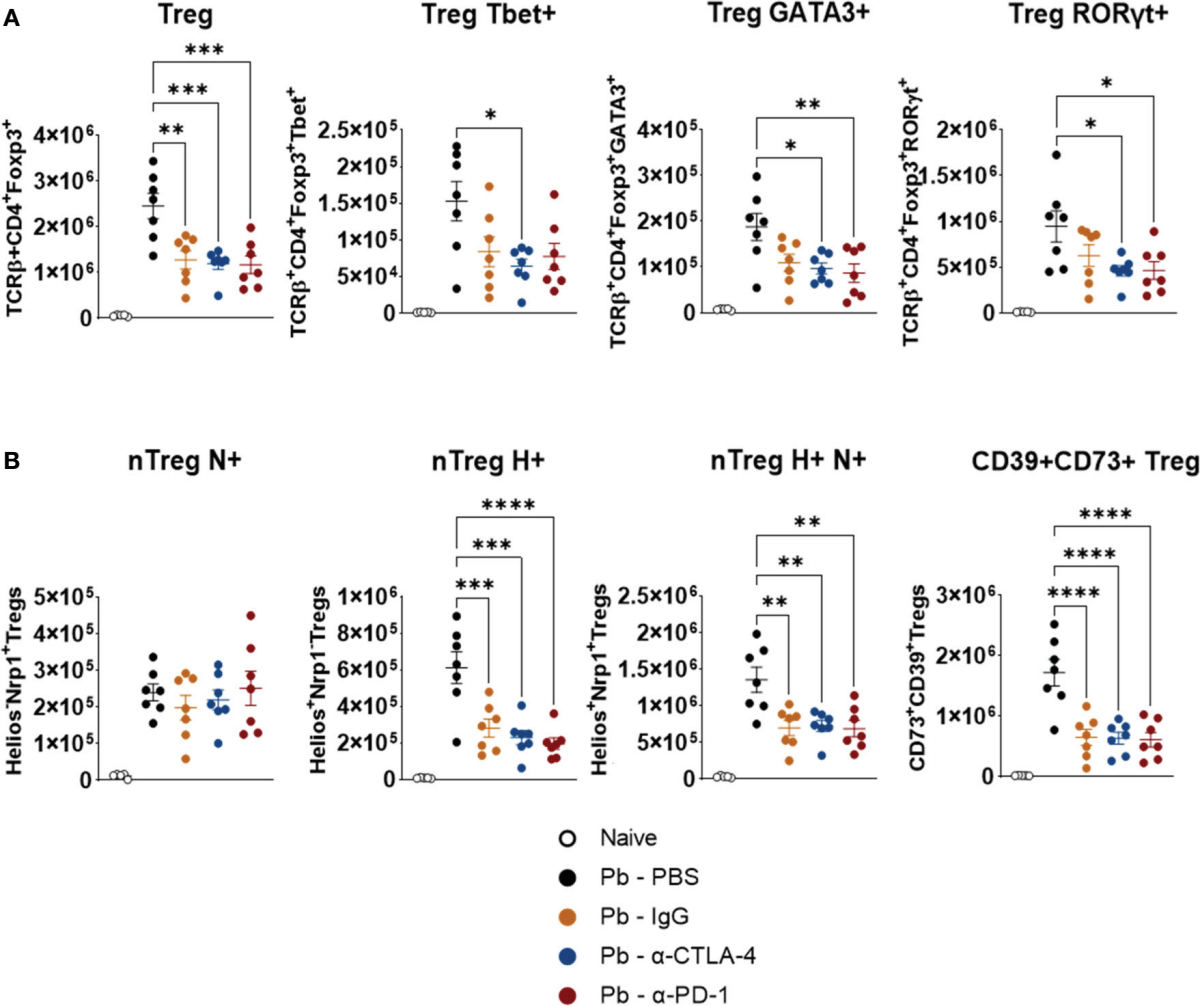
Compared with control Pb-PBS, blockade of checkpoint molecules reduces pulmonary infiltrates of Treg cells in *P. brasiliensis* infected mice. Number of Treg (TCD4+ Foxp3+) cells expressing the transcription factors Tbet, GATA3, and RORγτ **(A)**, and number of natural Treg (nTreg) cells expressing the maturation marker Neuropilin (N), the transcription factor Helios (H), and markers suppressive activity (ectonucleotidases CD39 and CD73) **(B)** present in the lungs of mice at the 8^th^ week post-infection with *P. brasiliensis* and treated in the course of the disease with α-CTLA-4, α-PD-1, control IgG mAbs or PBS. Experiments were repeated twice using 4-7 mice per group. Differences were considered significant when: *p<0.05; **p<0.01; ***p<0.001 and ****p<0.0001.

No significant differences were observed between the Pb-IgG and α-CPI-treated groups. However, compared with the PBS control, α-CTLA-4 treatment reduces the number of pulmonary Tregs expressing the transcription factors Tbet, GATA3, and RORγτ. Moreover, PD-1 blockade diminished GATA3+ and RORγτ+ Tregs (**Figure 5A**). Thus, Pb infection induces the expression of Th1, Th2, and Th17 transcription factors by Tregs, possibly reflecting the expansion of mixed T-cell subpopulations. In contrast, mAb treatments caused a significant reduction in these Treg phenotypes. CTLA-4 and PD-1 blockade, as well as control IgG treatment, reduced the presence of natural Tregs expressing Helios and Neuropilin, in addition to significantly reducing the number of Treg+CD39+CD73+ cells, which are highly suppressive (**Figure 5B**). **Supplementary Figure 1** shows the gating strategy used to analyze the expression of maturation, differentiation, and suppression markers (Tbet, GATA3, RORγτ, Helios, Neuropilin-1, CD39, and CD73) by pulmonary Treg lymphocytes from mice.

### 3.5 Blockade of CTLA-4, PD-1 (and control IgG) reduces pulmonary infiltration of several myeloid cell subpopulations, the expression of costimulatory molecules (CD40, CD80, and CD86), and the expression of the enzyme indoleamine dioxygenase (IDO)

**Figure 6:**
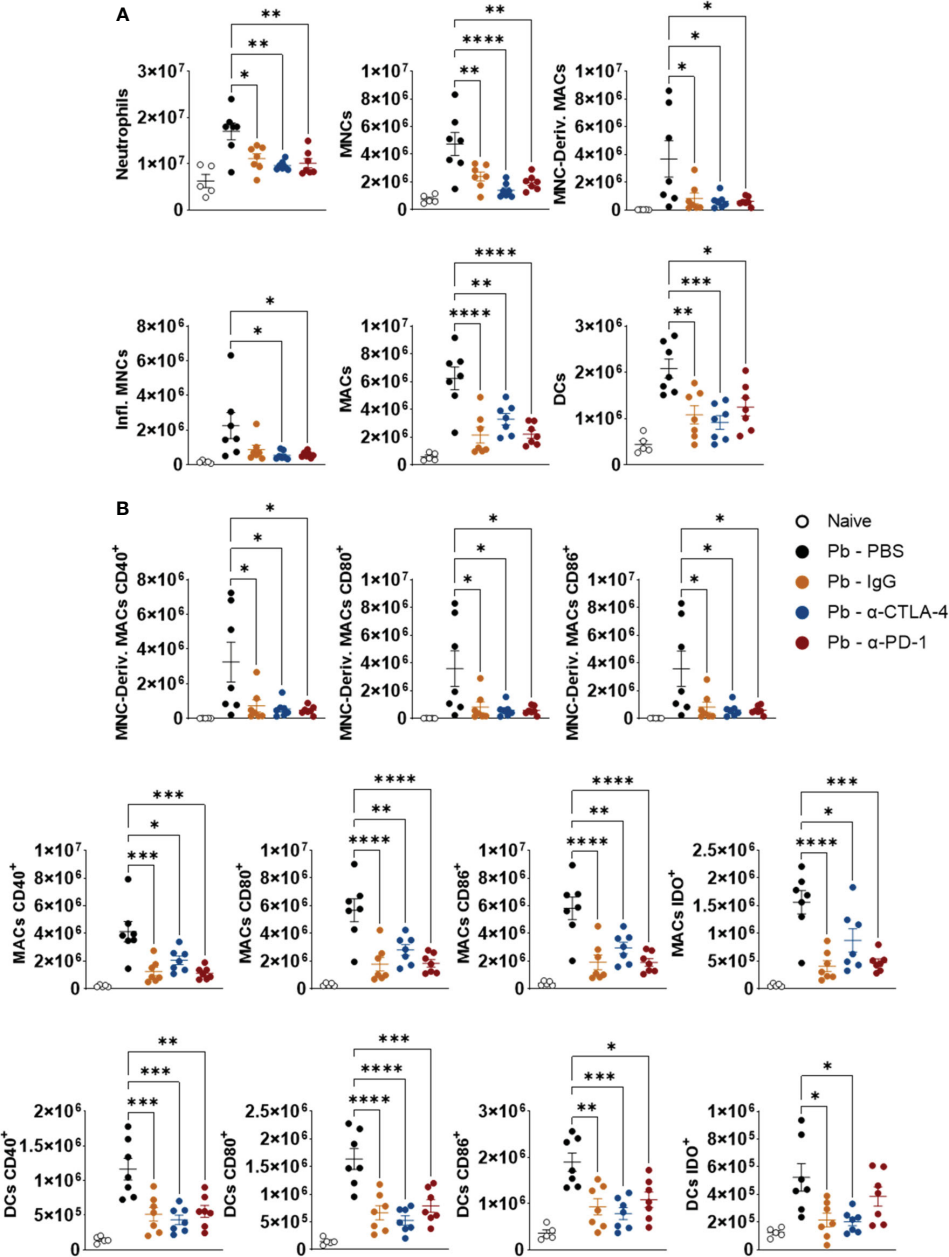
Compared to the Pb-PBS control, blockade of CTLA-4 and PD-1 reduces the presence of several subpopulations of lung-infiltrating myeloid cells and their expression of costimulatory molecules (CD40, CD80, CD86) and the enzyme IDO. Number of pulmonary Neutrophils (CD11b+ Ly6G+), Monocytes (MNCs, CD11b+ LY6C+ MHC II-), Monocyte-Derived Macrophages (MNC-Deriv. MACs, CD11b+Ly6C+ F4/80+), Inflammatory Monocytes (Infl. MNCs, C11b+ Ly6C+ MHCII+), Macrophages (MACs, CD11b+ F4/80+) and Dendritic Cells (DCs, CD11c+ MHCII+) **(A)**; Monocyte-Derived Macrophages, Macrophages, and Dendritic Cells expressing activation molecules (CD40, CD80, CD86) and the enzyme Indoleamine Dioxygenase (IDO) **(B)** present in the lungs of mice at the 8^th^ week after *P. brasiliensis* infection and treated in the course of the disease with α-CTLA-4, α-PD1, control IgG mAbs or PBS for five weeks. Experiments were repeated twice using 4-7 mice per group Differences were considered significant when: *p<0.05; **p<0.01; ***p<0.001 and ****p<0.0001.

**Figure 6A** shows that, compared to the Pb-PBS control, blockade of CTLA-4 and PD-1, as well as the IgG control, reduced the number of lung neutrophils (CD11b+ Ly6G+), monocytes (CD11b+Ly6C+MHC II-), macrophage-derived monocytes (CD11b+Ly6C+ F4/80+), inflammatory monocytes (C11b+ Ly6C+ MHCII+), macrophages (CD11b+ F4/80+) and dendritic cells (DCs, CD11c+ MHCII+) (**Figure 6A**). Treatment with all the mAbs also significantly reduced the expression of CD40, CD80, CD86 and IDO by monocyte-derived macrophages, macrophages and dendritic cells. (**Figure 6B**). **Supplementary Figure 3** shows the gating strategy used to characterize these myeloid cell subsets and their expression of CD40, CD80, CD86, and IDO by lung leukocytes from mice in the 8^th^ week post infection with *P. brasiliensis*.

### 3.6 Treatment with α-CPI mAbs exerts a minor influence on the expression of intracellular cytokines by CD45+ lung leukocytes

**Figure 7:**
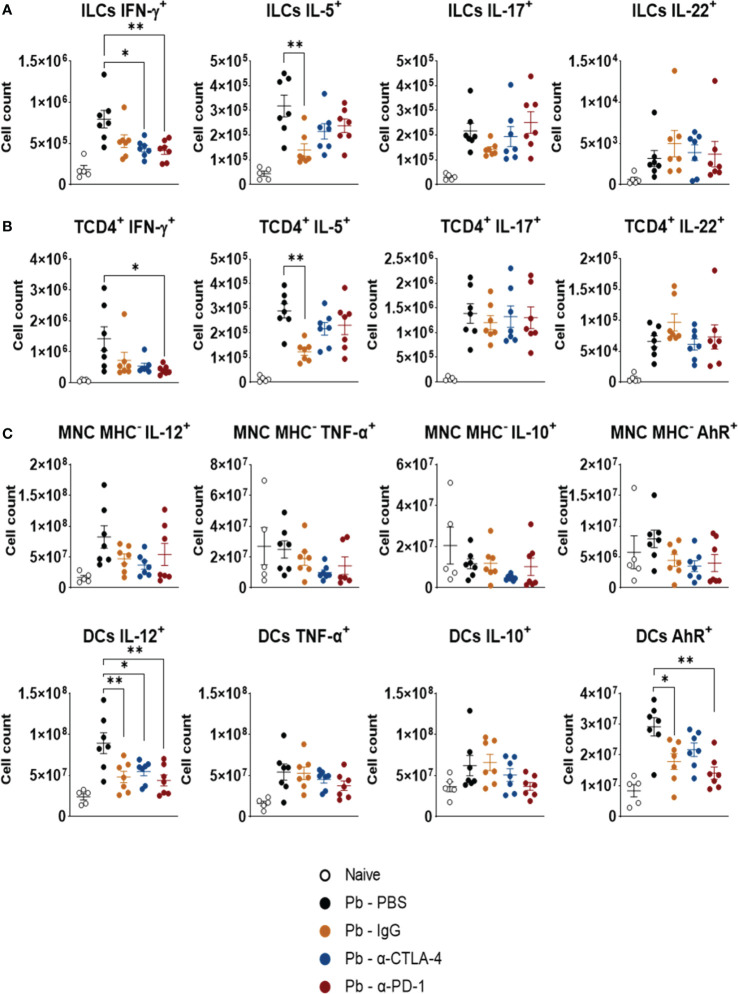
Expression of intracellular cytokines by CD45+ lung leukocytes. The presence of intracellular IFN-γ, IL-5, IL-17, and IL-22 was characterized in ILCs **(A)**, and TCD4+ lymphocyte subsets **(B)**. Expression of cytokines (IL-12, TNF-α, and IL-10), and the transcription factor AhR by monocytes (Ly11b+Ly6C+MHCII-), and dendritic cells (CD11c+ Gr1-) **(C)** present in the lungs of *P. brasiliensis* infected mice treated with α-CTLA-4 and α-PD-1 mAbs and their controls (Pb-PBS and Pb-IgG). Experiments were repeated twice using 4-7 mice per group. Differences were considered significant when: *p<0.05; **p<0.01.

**Figure 7** shows the expression of intracellular cytokines by ILCs (**Figure 7A**), TCD4+ lymphocytes (**Figure 7B**), monocytes, and DCs (**Figure 7C**). The control IgG exerted effects similar to those induced by α-CPI blockade. Compared with Pb-PBS, PD-1 blockade reduced the expression of IFN-γ by ILCs and TCD4+ cells. It also reduced the expression of IL-12 and AhR by DCs. Treatment with α CTLA-4 decreased only the expression of IFN-γ by ILCs and that of IL-12 by DCs. The gating strategy used to analyze these cells is shown in **Supplementary Figure 4**.

### 3.7 Blocking CTLA-4 and PD-1 (as well as control IgG) reduces the expression of costimulatory (ICOS, OX40L, and GITR) and coinhibitory (CTLA-4, PD-1, PD-L1, and CD39) molecules by Treg, TCD8+ and TCD4+ cells

**Figure 8:**
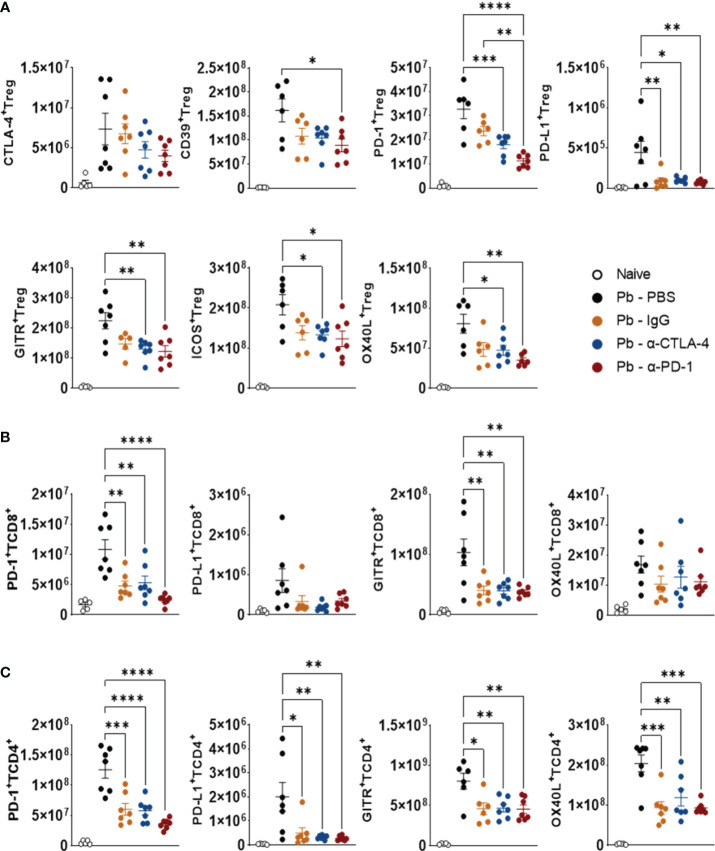
Compared with Pb-PBS control, blockade of CTLA-4 and PD-1 reduces the expression of costimulatory (ICOS, OX40L, GITR) and inhibitory (CTLA-4, PD-1, PD-L1, CD39) molecules by Treg, TCD8+ and TCD4+ cells. Treg (CD4+ Foxp3+) **(A)**, TCD8+ **(B)**, and TCD4+ **(C)** cells expressing suppressor molecules (CD39, CTLA-4, PD-1, and PDL-1) or co-stimulatory molecules (GITR, ICOS, and OX40L) present in the lungs of mice at the 8^th^ week post-infection with 1 x 10^6^ P*. brasiliensis* yeasts and treated in the course of the disease (from the 3rd week p.i. on) with α-CTLA-4 and α-PD-1, control IgG mAbs or PBS for 5 weeks. Differences were considered significant when: *p<0.05; **p<0.01; ***p<0.001 and ****p<0.0001.

As shown in **Figure 8**, compared with the Pb-PBS control, CTLA-4 inhibition reduced the presence of PD-1+, PDL-1+, GITR+, ICOS+, and OX40L+ Tregs in the lungs of *P. brasiliensis*-infected mice. This blockade also caused a reduction in the number of TCD4+ and TCD8+ T lymphocytes expressing PD-1 and GITR, in addition to TCD4+ expressing OX40L and PD-L1 (**Figures 8B, C**).

PD-1 blockade reduced the expression of all costimulatory and coinhibitory molecules, except CTLA-4, by Treg cells. This treatment also reduced the number of pulmonary TCD4+ and TCD8+ populations expressing PD-1 and GITR, while only TCD4+ T lymphocytes had reduced PD-L1 and OX40L levels (**Figures 8B, C**). The gating strategy used to characterize the expression of coinhibitory and costimulatory molecules by Tregs (CD4+Foxp3+), TCD4+, and TCD8+ lymphocytes in the lungs of *P. brasiliensis*-infected mice treated with α-CTLA-4, α-PD-1, control IgG mAbs and PBS is shown in **Supplementary Figure 5**.

## 4 Discussion

In chronic infections, persistent antigenic stimulation can lead to immunological exhaustion in a process similar to that of neoplasms. This approach has been used to propose the treatment of some chronic infectious diseases with immune checkpoint inhibitors.

Elevated expression of PD-1 on CD8+ and CD4+ T lymphocytes and exhaustion of these cells have been reported in several viral infections, such as lymphocytic choriomeningitis, AIDS, hepatitis B and C (40, 41). In addition to viral infections, other chronic infections induce exhaustion of the immune system, as reported for malaria (42) and tuberculosis (43). In these diseases, blocking CPI molecules can benefit the patient by restoring the immune response but can also lead to more severe illnesses due to exacerbated inflammatory processes (44).

Several studies have also demonstrated that anti-CPI treatment can alter the course of fungal diseases. For example, *Histoplasma capsulatum* induces the expression of PD-1 in macrophages, which inhibits the proliferation of T lymphocytes. The absence of PD-1 expression or its blockade leads to less severe disease in a murine model of the disease (45). In experimental models of candidiasis and cryptococcosis, treatment with α-PD-1 and α-CTLA-4 resulted in less severe disease, with lower fungal loads and a reinvigorated immune response (46, 47). However, the development of “immune reconstitution inflammatory syndrome” (IRIS) was described in patients with cryptococcosis with an exacerbated Th1 response (48). A recent study of experimental pulmonary aspergillosis demonstrated the protective effect of α-PD-1, which synergized with the antifungal drug caspofungin (12).

It is well documented that the immunosuppressive mechanisms in humans and experimental PCM are associated with the expression of coinhibitory molecules such as PD-1 and CTLA-4. These findings led us to postulate that blocking checkpoint inhibitory molecules could restore the protective effector mechanisms of the immune response. Indeed, the findings presented here demonstrate that both α-PD-1 and α-CTLA-4 treatments can control the number of fungi in the lungs and their dissemination to the liver and spleen. Furthermore, these agents reduce the size of lung lesions and mouse mortality.

CTLA-4 and PD-1 were blocked through repeated inoculation of two mAbs, α-CTLA-4, a hamster IgG, and α-PD-1, a rat IgG2a. To better control the disturbances that the repeated inoculation of a heterologous Ig could cause in the immune system, we chose to use two experimental controls, a nonspecific rat monoclonal IgG2a (Pb-IgG) and the repeated inoculation of PBS in infected animals (Pb-PBS). The immune-related adverse effects (irAEs) caused by α-CPI therapy are well known, even when humanized antibodies are used (49). As expected, in our study, heterologous IgG caused marked changes in the presence of cytokines in the organs and cell migration to the lungs of infected mice. Control IgG, however, did not interfere with disease severity. A similar result was reported by Roussey et al. (2016), who studied the effect of PD-1 blockade on pulmonary cryptococcosis (47). Despite the reduced fungal loads, no differences in the influx of several leukocyte subsets to the lungs were detected between α-PD-1-treated and control IgG-treated mice, which was also observed in our studies. Despite the similar cellular influx caused by control IgG, it is tempting to suggest that the protective effect of α-CPI treatment was due to the reduced number of Treg cells combined with the increased activation of Pb-specific cells, a phenomenon not induced by the repeated inoculation of nonspecific heterologous IgG. In addition, using Pb-PBS as a control allowed us to better follow the immune disturbances caused by heterologous Ig and bring our results closer to what happens in human immunotherapy where α-CPI antibodies are humanized.

We can propose several hypotheses to explain the influence of a heterologous Ig in the immune response. The low level of LPS contamination in rat IgG may have induced an inflammatory process due to the repeated inoculations. On the other hand, the regulatory effects on the immune system mediated by the idiotypic immunoglobulin network are well known and may have been disrupted by the inoculation of control IgG (50–52). More importantly, the repeated inoculation of control IgG into infected animals may have altered the behavior of cells harboring Fcγ receptors, such as macrophages, monocytes, neutrophils, DCs, and B lymphocytes. It is also worth noting that repeated inoculation of a control IgG may have induced the production of anti-IgG antibodies and the development of immune complexes that activate the complement system and could have induced systemic vasculitis by immune complex deposition. Although not addressed here, the inflammatory disturbances caused by heterologous IgG deserve further investigation.

Importantly, our data are snapshots of the immune response after five weeks of treatment, which possibly underwent several changes during this period. Despite this, our results indicate the development of a more efficient immune response capable of controlling the growth and dissemination of the fungus without apparent deleterious effects.

Compared with controls, important differences in the levels of pulmonary and hepatic cytokines were observed after blockade of both CPIs. In the lungs, CTLA-4 blockade led to increased levels of pro- and anti-inflammatory cytokines, which are involved in Th17 (IL-23, IL-17, and IL-22), Th1 (IL-12, IL-2, and GM-CSF), and Th2 (IL-10, IL-4 and IL-5) responses. These findings mimic the benign forms of human and experimental PCM (13, 24, 25, 33) and may have contributed to the control of fungal loads and increased survival of α-CTLA-4-treated mice. PD-1 blockade exerted a less important effect on pulmonary cytokines but a striking reduction in liver cytokine levels, detected when the Pb-PBS group was used as control. These alterations appear to have contributed to the protective effect of PD-1 blockade in pulmonary PCM.

Interestingly, CTLA-4 and PD-1 blockade resulted in diverse lung and liver cytokine patterns that were possibly influenced by the main inhibitory mechanisms used by these checkpoint molecules. The CTLA-4 pathway is predominantly involved in the activation of naïve T cells at the priming stage, inhibiting the interaction of CD28 with CD80 and CD86 due to its increased affinity (3, 5), and possibly mainly suppressed T-cell activation in the lungs, the site of *P. brasiliensis* inoculation. PD-1 is widely expressed by T cells, B cells, natural killer (NK) cells, and mononuclear phagocytes (3, 9), and the reduced fungal dissemination to the liver caused by PD-1 blockade possibly inhibits the migration and release of cytokines by all these effector leukocytes. In addition, the differences in cytokine patterns between the lungs and liver could also be attributed to the predominant tolerogenic environment of the liver, which is essential for preventing excessive immune responses induced by the influx of food debris and microorganisms through the hepatic portal vein (53, 54). Thus, after α-CPI treatments, which enables a more efficient immune response that reduces the hepatic fungal load, the liver possibly returns to its tolerogenic default state, inhibiting excessive inflammatory processes.

Analysis of lung cell populations by tSNE showed that blockade of CTLA-4 and PD-1 increased the migration of CD45+ leukocytes to the lung parenchyma and reduced the number of CD45+ cells from the vasculature. The frequency of almost all cell phenotypes was not altered by the employed treatments, except for the increased frequency of ILC1 NK cells in the Pb-α-CTLA-4 group. In contrast, the total number of CD45+ leukocytes was reduced in all the treated groups.

Treatment with anti-CPI mAbs and control IgG significantly reduced the total numbers of TCD4+ and TCD8+ lymphocytes. Among the CD4+ T cells, Th1, Th2, and Th17 lymphocytes were maintained, but there was an important reduction in the Treg cells with various phenotypes, such as those expressing RORγτ, GATA3, or Tbet. These groups of Treg cells, which express the transcription factors of effector T-cell subpopulations, Th17, Th2, and Th1, respectively, were demonstrated for the first time in pulmonary PCM. It is debated whether these Treg cell subsets represent stable populations or even intermediate populations with dual regulatory/effector activity; however, functionally, these subpopulations are compromised with tissue suppression of their corresponding Th subpopulation due to similar migration processes through the typical expression of chemokine receptors (55, 56). In addition, the number of CD25+ Treg cells and those expressing the suppressive ectoenzymes CD39 and CD73 were also diminished in the lungs of α-CPI-treated mice. These data indicate that the reduction in pulmonary CD4+ T cells did not occur at the expense of effector phenotypes but rather occurred through a significant reduction in Treg cells in the inflammatory infiltrate of the lungs. This reduction in Treg infiltrates indicates increased effector activity of the immune response in the lungs of α-CPI-treated mice, which could control fungal growth and dissemination but not in control IgG-treated mice, where no differences in disease severity were found. These findings corroborate those reported by Felonato et al. (2012) and Bazan et al. (2015) in experimental PCM and by Cavassani et al. (2006), Cardoso et al. (2014) and Ferreira et al. (2010) in human PCM, where a less severe disease is associated with lower numbers of Treg cells in lesions or peripheral blood (19–21, 23, 24).

The analysis of the differentiation and activation of the TCD4+ and Treg Foxp3+ subpopulations demonstrated the maintenance of the naïve, effector/memory, and CD25+TCD4+ subpopulations, with a concomitant reduction in central memory TCD4+ cells. The maintenance of the number of effector/memory CD4+ T cells and activated CD4+ T cells suggested a more efficient cellular response capable of reducing the pathogen load without causing harmful inflammatory reactions in the lungs. A reduction of Central Memory TCD4+ cells is a common finding in studies of the cellular composition of tumor-infiltrating leukocytes after treatment with checkpoint inhibitors (57). However, there is a very promising treatment for advanced breast tumors (hormone receptor-positive) with the drug CDK4/6, a cyclin-dependent kinase inhibitor that restores the immune response (58, 59). Blocking PD-1 concomitantly with the administration of CDK4/6 has a synergistic protective effect against tumors by restoring T effector populations and promoting the formation of central memory T cells that preserve the immune-activating effect of α-CPI treatment (57). Studying the joint blockade of checkpoint molecules and drugs that stimulate central memory T cells in pulmonary PCM would be interesting.

A striking result of the blockade of CPI molecules was the decrease in the number of Treg cells expressing Helios, Neuropilin, and the suppressive ectoenzymes CD39 and CD73. These data are in agreement with a recent publication that demonstrated, through single-cell mRNAseq analysis, that the inhibition or elimination of PD-1 molecules results in the inhibition of proliferation and suppression of Treg cells by destabilizing the expression of Foxp3 and altering lipid metabolism (60). Furthermore, this decrease in Tregs was associated with decreased expression of the enzyme IDO and the transcription factor AhR in some cell populations, corroborating our studies demonstrating the importance of these molecules in the differentiation of Treg cells and the severity of PCM (30, 31, 33).

Another finding that corroborates the greater efficiency of the immune response after CTLA-4 and PD-1 blockade was the maintenance of several populations of ILCs, TCD4+, and TCD8 cells expressing intracellular cytokines despite the reduction in fungal load. The same is true for lung-infiltrating monocytes and DCs expressing intracellular cytokines. These observations suggest that the increase in pulmonary cytokines observed in the lungs was mainly due to increased activation and effector function of T cells.

The reduction in fungal burden induced by CPI blockade caused diminished inflammation associated with a reduced presence of myeloid cells in the lungs (neutrophils, monocytes, inflammatory monocytes, monocyte-derived macrophages, macrophages, and DCs). Interestingly, costimulatory molecules (CD40, CD80, and CD86) and the enzyme IDO (a Treg cell inducer in murine PCM) were reduced by myeloid cells that migrated to the lungs after CTLA-4 and PD-1 inhibition. Therefore, the more efficient immunity of α-CPI-treated mice was not associated with increased migration of inflammatory cells, which apparently has not caused harmful problems in the infected tissue.

The inhibition of checkpoint molecules led to a new activation profile of CD4+ T cells, CD8+ T cells, and Treg Foxp3+ lymphocytes. Both treatments caused a decrease in the expression of costimulatory molecules (ICOS, OX40L, and GITR) and coinhibitory molecules (PD-1 and PD-L1) induced by *P. brasiliensis* infection. This finding was associated with a decreased presence of fungal cells, which possibly induced reduced costimulatory activity in immune cells and subsequent control of immunity mediated by coinhibitory molecules.

In summary, blocking the CTLA-4 and PD-1 checkpoint molecules that inhibit the immune response during pulmonary PCM reduces the severity of the disease and increases host survival. The immune response established in the lungs showed increased levels of pro- and anti-inflammatory cytokines, possibly produced by TCD4+ and TCD8+ cells. The numbers of Th1, Th2, and Th17 lymphocytes were maintained, but several Treg cell subpopulations and their effector mechanisms were reduced. In addition, a decreased influx and activation of myeloid cells were detected in the lungs, as was the expression of costimulatory and coinhibitory molecules by T cells. The new cellular profile established in the lungs showed more efficient effector activity and was able to control the growth and dissemination of the fungus without causing an increase in tissue pathology due to an excessive inflammatory process.

Despite these encouraging results, our study has several limitations. Therefore, dose-response studies with different anti-CPI monoclonal agents, such as α-PD-L1, α−TIM3, α-TIGIT, and evaluations of different treatment protocols for changing the frequency and duration of antibody administration are necessary. Studies should also be carried out using homologous antibodies to avoid disorders caused by the immune response against heterologous proteins. Furthermore, the association between α-CPI therapy and antifungal drugs should also be studied, aiming for a possible synergistic effect to protect patients from this serious endemic mycosis.

Reports on the expression of checkpoint molecules and their importance in the control and therapy of fungal infections are scarce, and additional studies are necessary to improve the efficacy of therapeutic processes for reducing patient suffering. We believe that our first study aimed to evaluate the effect two CPI molecules blockade opened new therapeutic options for pulmonary PCM and should be further explored.

## Data availability statement

The raw data supporting the conclusions of this article will be made available by the authors, without undue reservation.

## Ethics statement

The animal study was approved by Committee on Animal Experiments of the Institute of Biomedical Sciences of the University of São Paulo. The study was conducted in accordance with the local legislation and institutional requirements.

## Author contributions

NP: Data curation, Formal analysis, Investigation, Supervision, Validation, Visualization, Writing – review & editing. BB: Data curation, Formal analysis, Investigation, Writing – review & editing. VK: Formal analysis, Investigation, Writing – review & editing, Data curation. MA: Formal analysis, Investigation, Writing – review & editing, Data curation. LG: Formal analysis, Writing – review & editing, Investigation. BS: Writing – review & editing, Investigation. DLF: Data curation, Formal analysis, Writing – review & editing. IF: Writing – review & editing, Formal analysis. CS: Formal analysis, Investigation, Writing – review & editing. SM: Formal analysis, Investigation, Writing – review & editing. OC-M: Formal analysis, Supervision, Validation, Writing – review & editing. DMF: Data curation, Formal analysis, Investigation, Methodology, Resources, Supervision, Validation, Writing – review & editing. FL: Investigation, Methodology, Resources, Supervision, Validation, Writing – original draft, Writing – review & editing. VC: Conceptualization, Data curation, Funding acquisition, Methodology, Project administration, Resources, Supervision, Validation, Visualization, Writing – original draft, Writing – review & editing.
