# Supplementary material for: Blocking the CTLA-4 and PD-1 pathways during pulmonary paracoccidioidomycosis improves immunity, reduces disease severity, and increases the survival of infected mice
Source: Front Immunol. 2024 Mar 4;15:1347318. doi: 10.3389/fimmu.2024.1347318 (PMC10945025; doi:10.3389/fimmu.2024.1347318)
Supplement: Supplementary file 1 [file Presentation_1.pptx]

## Slide 1
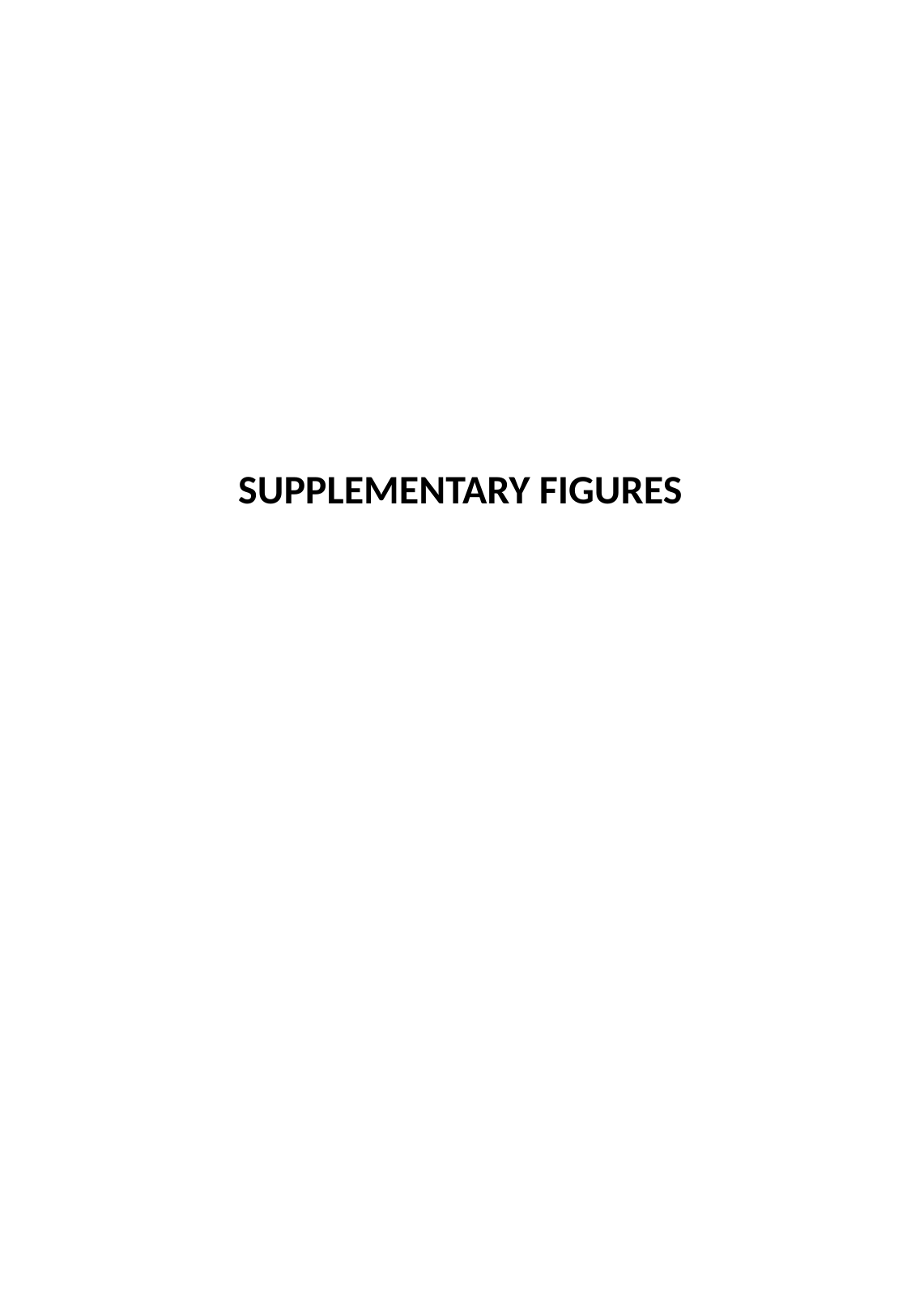

SUPPLEMENTARY FIGURES

## Slide 2
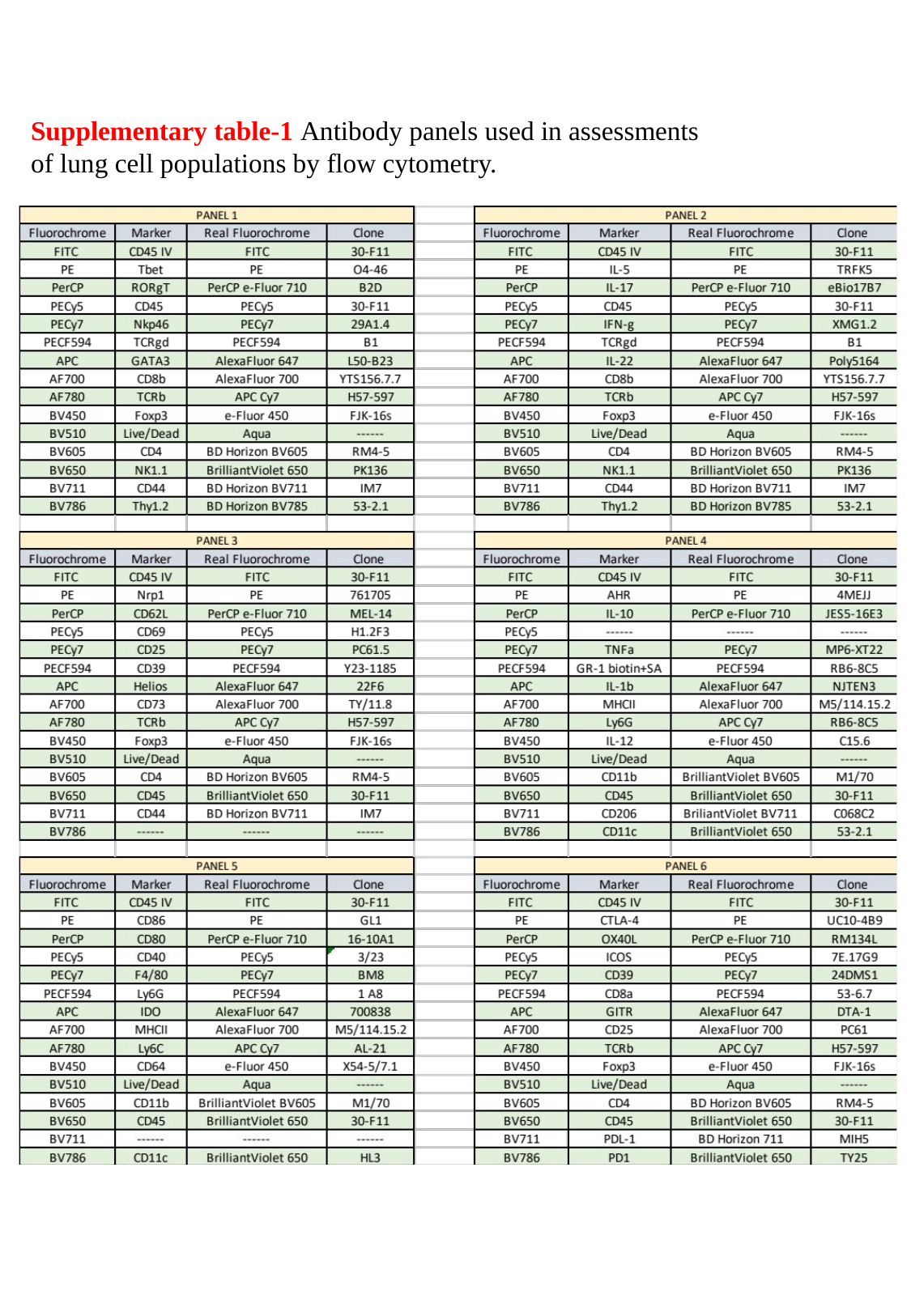

Supplementary table-1 Antibody panels used in assessments
of lung cell populations by flow cytometry.

## Slide 3
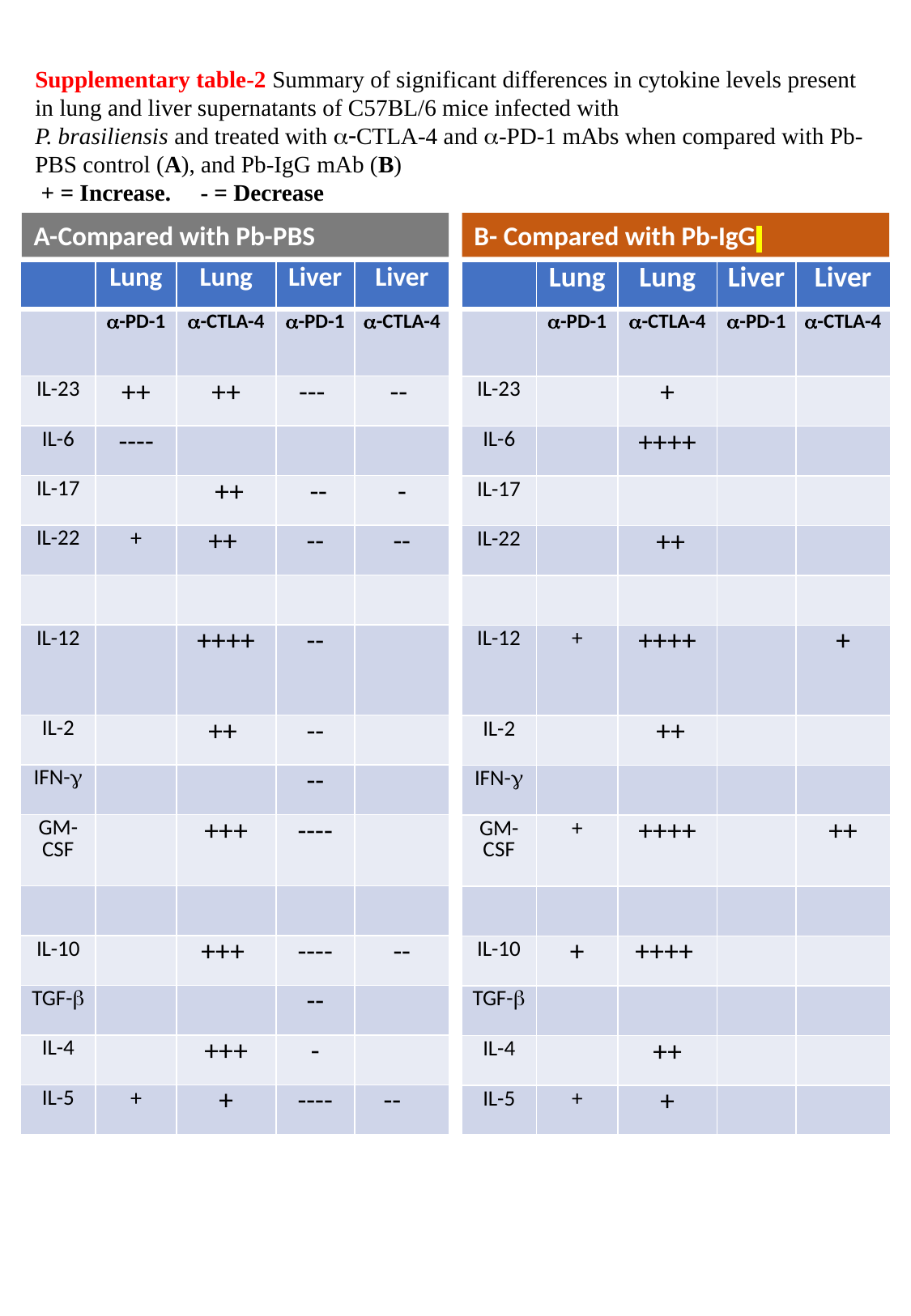

Supplementary table-2 Summary of significant differences in cytokine levels present in lung and liver supernatants of C57BL/6 mice infected with
P. brasiliensis and treated with a-CTLA-4 and a-PD-1 mAbs when compared with Pb-PBS control (A), and Pb-IgG mAb (B)
 + = Increase. - = Decrease
B- Compared with Pb-IgG
A-Compared with Pb-PBS
| | Lung | Lung | Liver | Liver |
| --- | --- | --- | --- | --- |
| | a-PD-1 | a-CTLA-4 | a-PD-1 | a-CTLA-4 |
| IL-23 | ++ | ++ | --- | -- |
| IL-6 | ---- | | | |
| IL-17 | | ++ | -- | - |
| IL-22 | + | ++ | -- | -- |
| | | | | |
| IL-12 | | ++++ | -- | |
| IL-2 | | ++ | -- | |
| IFN-g | | | -- | |
| GM-CSF | | +++ | ---- | |
| | | | | |
| IL-10 | | +++ | ---- | -- |
| TGF-b | | | -- | |
| IL-4 | | +++ | - | |
| IL-5 | + | + | ---- | -- |
| | Lung | Lung | Liver | Liver |
| --- | --- | --- | --- | --- |
| | a-PD-1 | a-CTLA-4 | a-PD-1 | a-CTLA-4 |
| IL-23 | | + | | |
| IL-6 | | ++++ | | |
| IL-17 | | | | |
| IL-22 | | ++ | | |
| | | | | |
| IL-12 | + | ++++ | | + |
| IL-2 | | ++ | | |
| IFN-g | | | | |
| GM-CSF | + | ++++ | | ++ |
| | | | | |
| IL-10 | + | ++++ | | |
| TGF-b | | | | |
| IL-4 | | ++ | | |
| IL-5 | + | + | | |

## Slide 4
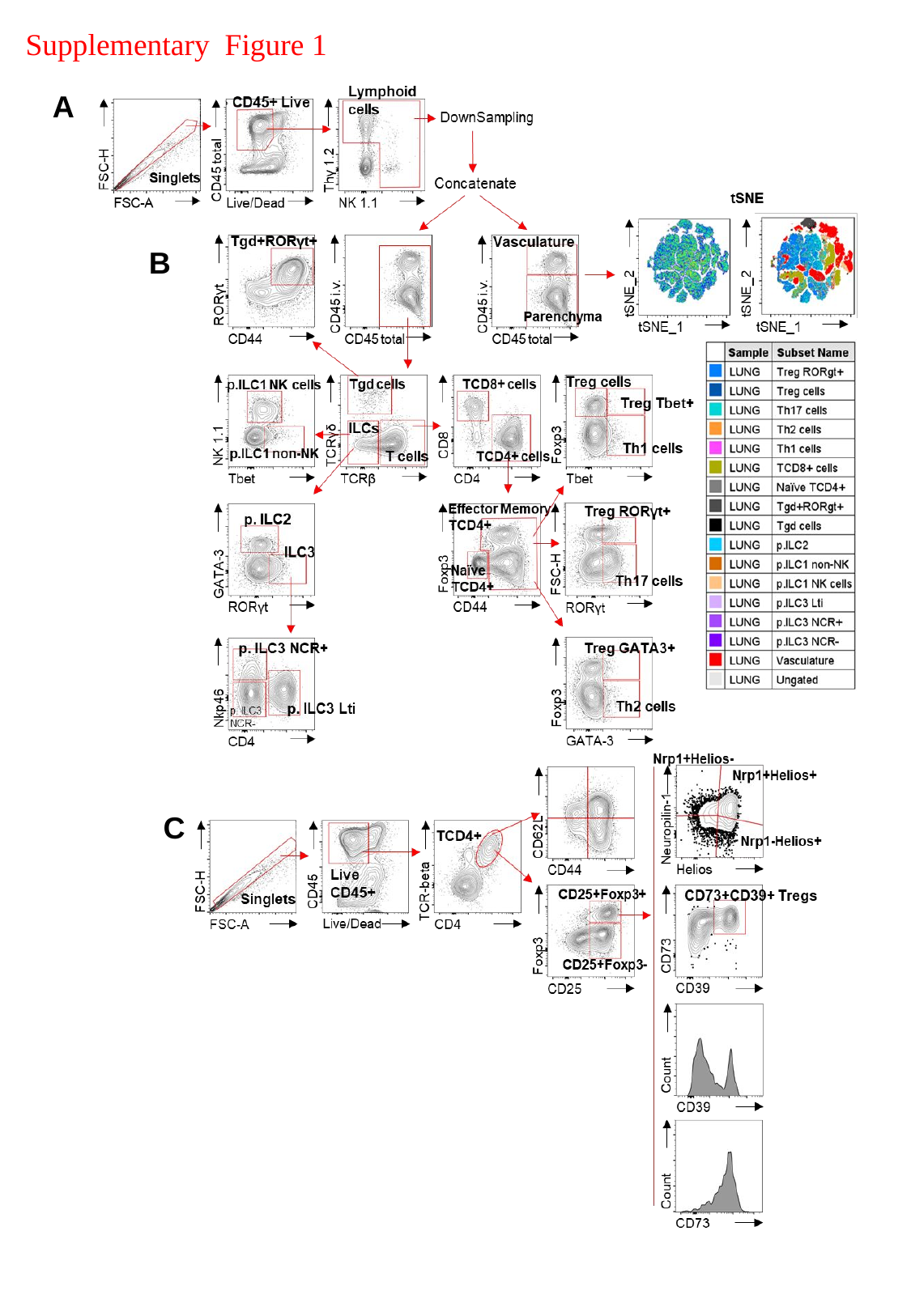

Supplementary Figure 1
A
B
C

## Slide 5
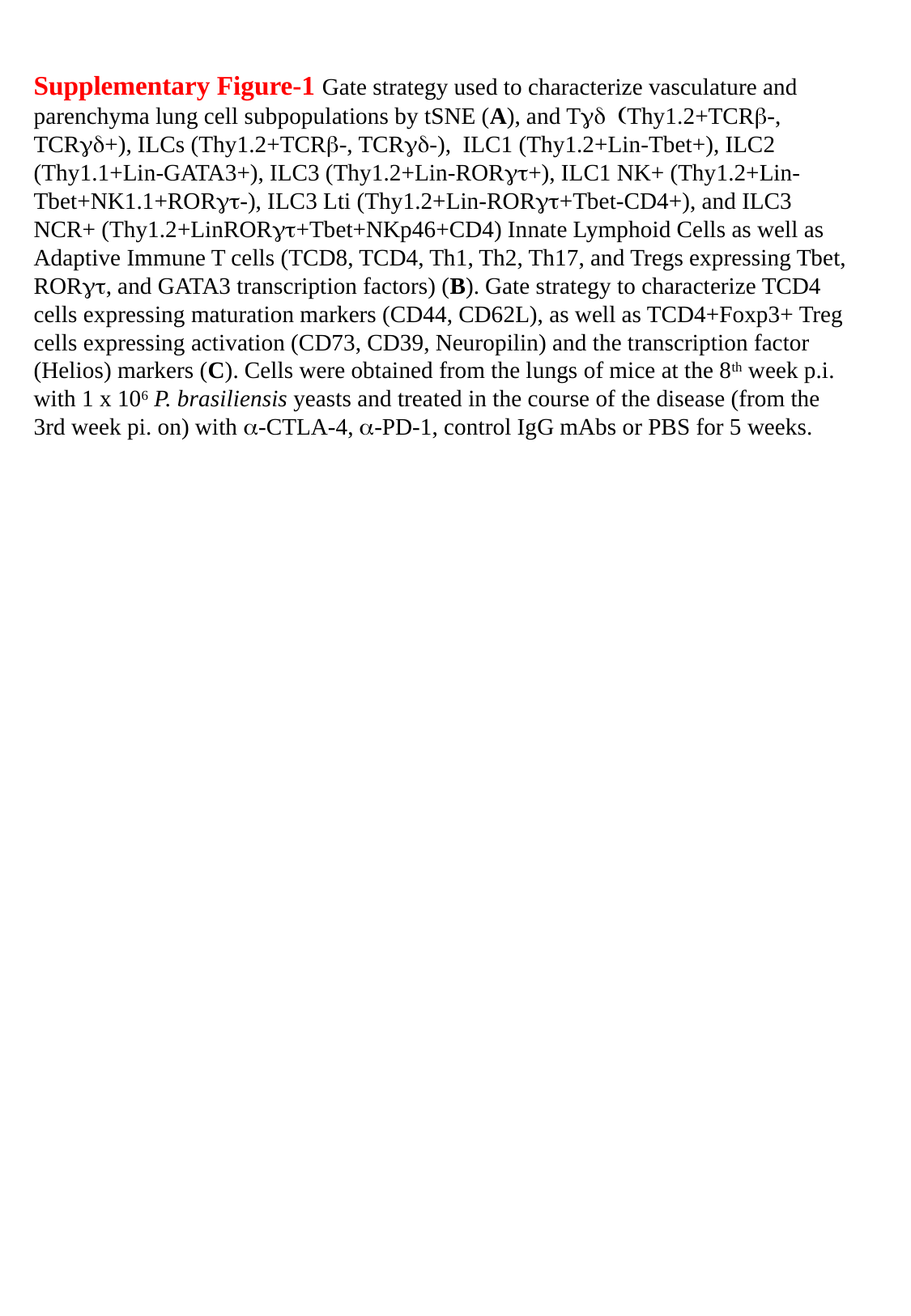

Supplementary Figure-1 Gate strategy used to characterize vasculature and parenchyma lung cell subpopulations by tSNE (A), and Tgd (Thy1.2+TCRb-, TCRgd+), ILCs (Thy1.2+TCRb-, TCRgd-), ILC1 (Thy1.2+Lin-Tbet+), ILC2 (Thy1.1+Lin-GATA3+), ILC3 (Thy1.2+Lin-RORgt+), ILC1 NK+ (Thy1.2+Lin-Tbet+NK1.1+RORgt-), ILC3 Lti (Thy1.2+Lin-RORgt+Tbet-CD4+), and ILC3 NCR+ (Thy1.2+LinRORgt+Tbet+NKp46+CD4) Innate Lymphoid Cells as well as Adaptive Immune T cells (TCD8, TCD4, Th1, Th2, Th17, and Tregs expressing Tbet, RORgt, and GATA3 transcription factors) (B). Gate strategy to characterize TCD4 cells expressing maturation markers (CD44, CD62L), as well as TCD4+Foxp3+ Treg cells expressing activation (CD73, CD39, Neuropilin) and the transcription factor (Helios) markers (C). Cells were obtained from the lungs of mice at the 8th week p.i. with 1 x 10^6^ P. brasiliensis yeasts and treated in the course of the disease (from the 3rd week pi. on) with a-CTLA-4, a-PD-1, control IgG mAbs or PBS for 5 weeks.

## Slide 6
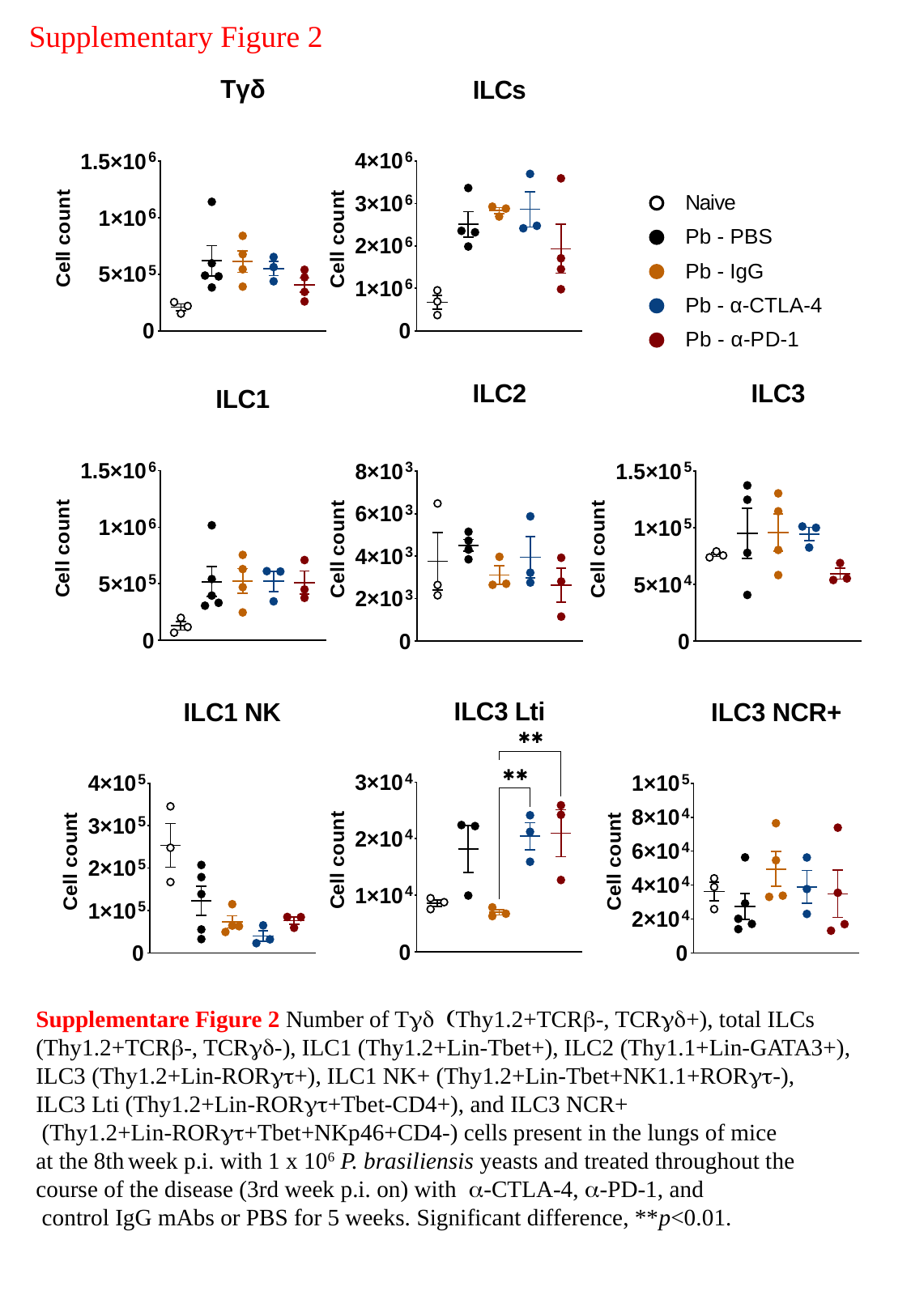

Supplementary Figure 2
Supplementare Figure 2 Number of Tgd (Thy1.2+TCRb-, TCRgd+), total ILCs
(Thy1.2+TCRb-, TCRgd-), ILC1 (Thy1.2+Lin-Tbet+), ILC2 (Thy1.1+Lin-GATA3+),
ILC3 (Thy1.2+Lin-RORgt+), ILC1 NK+ (Thy1.2+Lin-Tbet+NK1.1+RORgt-),
ILC3 Lti (Thy1.2+Lin-RORgt+Tbet-CD4+), and ILC3 NCR+
 (Thy1.2+Lin-RORgt+Tbet+NKp46+CD4-) cells present in the lungs of mice
at the 8th week p.i. with 1 x 10^6^ P. brasiliensis yeasts and treated throughout the
course of the disease (3rd week p.i. on) with a-CTLA-4, a-PD-1, and
 control IgG mAbs or PBS for 5 weeks. Significant difference, **p<0.01.

## Slide 7
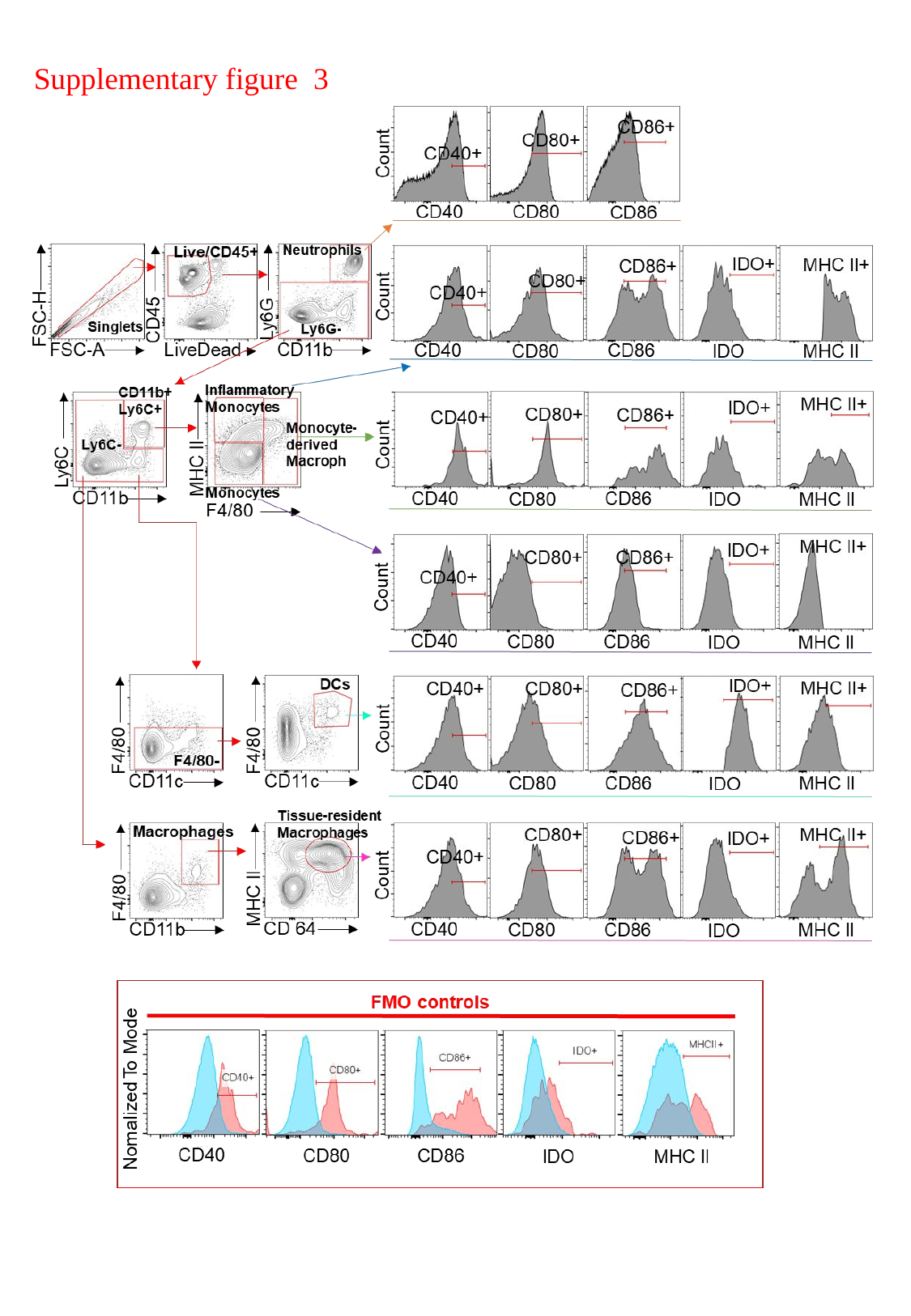

Supplementary figure 3

## Slide 8
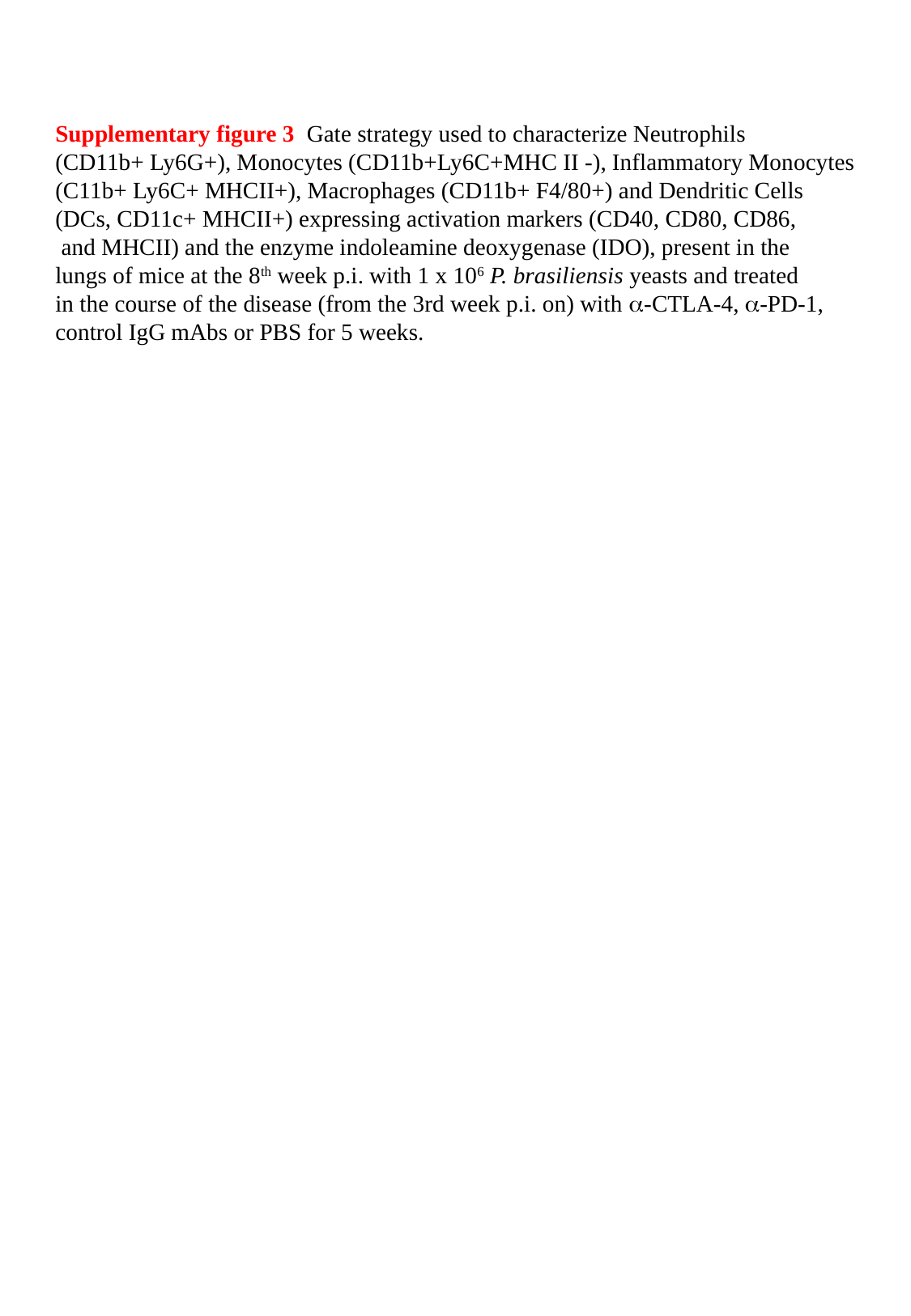

Supplementary figure 3 Gate strategy used to characterize Neutrophils
(CD11b+ Ly6G+), Monocytes (CD11b+Ly6C+MHC II -), Inflammatory Monocytes
(C11b+ Ly6C+ MHCII+), Macrophages (CD11b+ F4/80+) and Dendritic Cells
(DCs, CD11c+ MHCII+) expressing activation markers (CD40, CD80, CD86,
 and MHCII) and the enzyme indoleamine deoxygenase (IDO), present in the
lungs of mice at the 8th week p.i. with 1 x 10^6^ P. brasiliensis yeasts and treated
in the course of the disease (from the 3rd week p.i. on) with a-CTLA-4, a-PD-1,
control IgG mAbs or PBS for 5 weeks.

## Slide 9
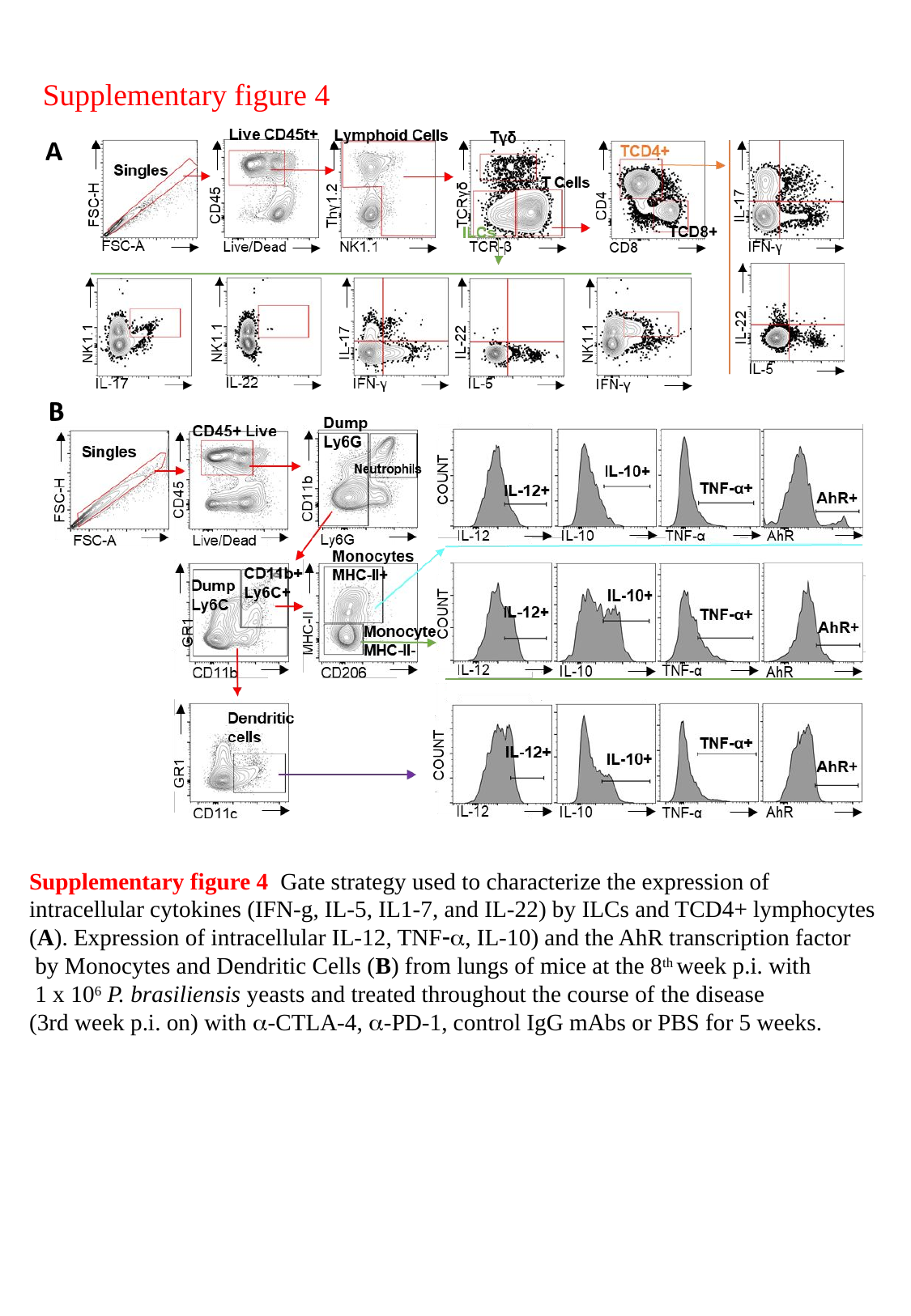

Supplementary figure 4
Supplementary figure 4 Gate strategy used to characterize the expression of
intracellular cytokines (IFN-g, IL-5, IL1-7, and IL-22) by ILCs and TCD4+ lymphocytes (A). Expression of intracellular IL-12, TNF-a, IL-10) and the AhR transcription factor
 by Monocytes and Dendritic Cells (B) from lungs of mice at the 8th week p.i. with
 1 x 10^6^ P. brasiliensis yeasts and treated throughout the course of the disease
(3rd week p.i. on) with a-CTLA-4, a-PD-1, control IgG mAbs or PBS for 5 weeks.

## Slide 10
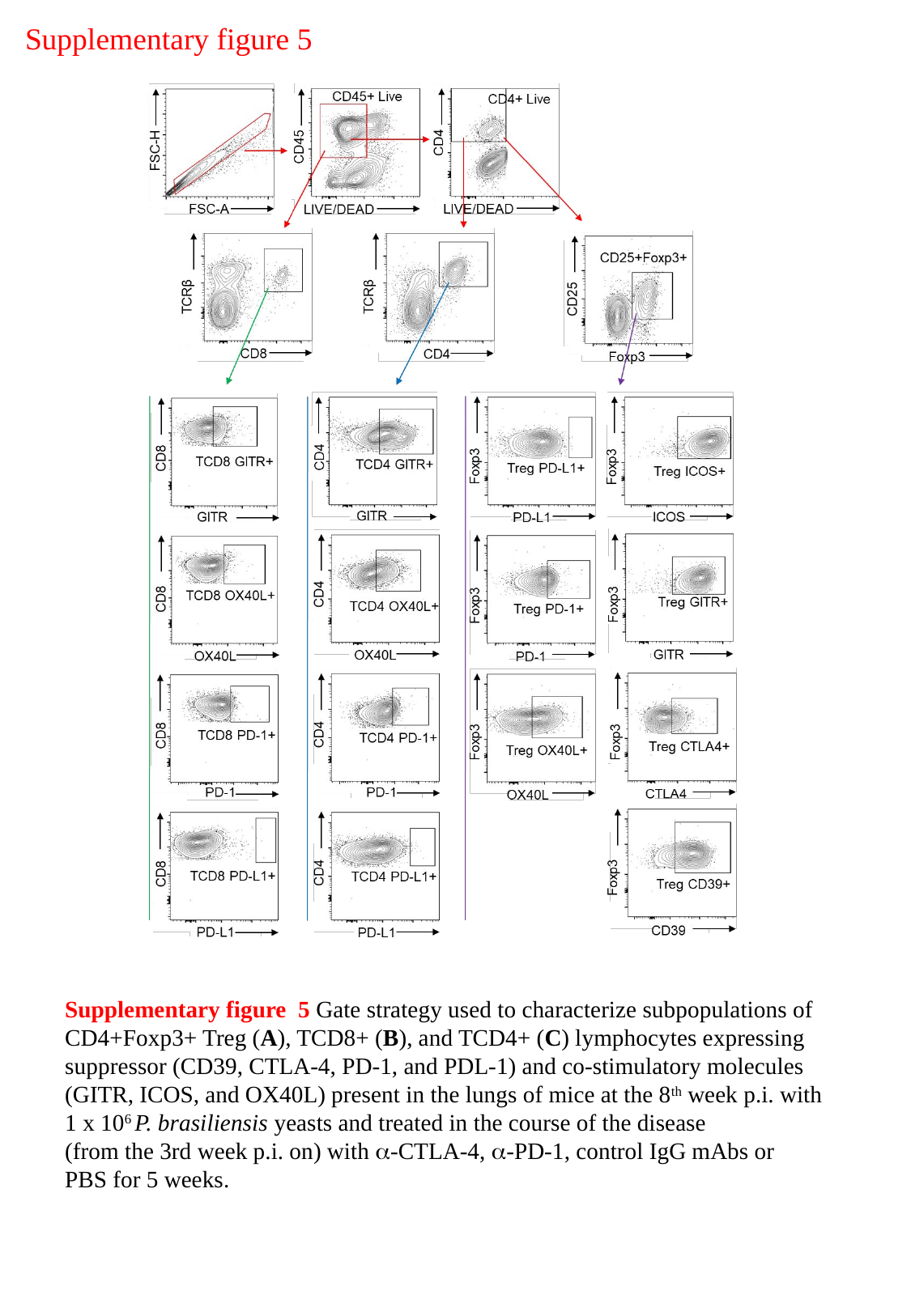

Supplementary figure 5
Supplementary figure 5 Gate strategy used to characterize subpopulations of
CD4+Foxp3+ Treg (A), TCD8+ (B), and TCD4+ (C) lymphocytes expressing
suppressor (CD39, CTLA-4, PD-1, and PDL-1) and co-stimulatory molecules
(GITR, ICOS, and OX40L) present in the lungs of mice at the 8th week p.i. with
1 x 10^6^ P. brasiliensis yeasts and treated in the course of the disease
(from the 3rd week p.i. on) with a-CTLA-4, a-PD-1, control IgG mAbs or
PBS for 5 weeks.
